# Thalamostriatal disconnection underpins long-term seizure freedom in frontal lobe epilepsy surgery

**DOI:** 10.1093/brain/awad085

**Published:** 2023-04-17

**Authors:** Davide Giampiccolo, Lawrence P Binding, Lorenzo Caciagli, Roman Rodionov, Chris Foulon, Jane de Tisi, Alejandro Granados, Roisin Finn, Debayan Dasgupta, Fenglai Xiao, Beate Diehl, Emma Torzillo, Jan Van Dijk, Peter N Taylor, Matthias Koepp, Andrew W McEvoy, Sallie Baxendale, Fahmida Chowdhury, John S Duncan, Anna Miserocchi

**Affiliations:** Department of Clinical and Experimental Epilepsy, UCL Queen Square Institute of Neurology, University College London, London WC1N 3BG, UK; Victor Horsley Department of Neurosurgery, National Hospital for Neurology and Neurosurgery, London WC1N 3BG, UK; Institute of Neuroscience, Cleveland Clinic London, London SW1X 7HY, UK; Department of Clinical and Experimental Epilepsy, UCL Queen Square Institute of Neurology, University College London, London WC1N 3BG, UK; Department of Computer Science, Centre for Medical Image Computing, University College London, London WC1V 6LJ, UK; Department of Clinical and Experimental Epilepsy, UCL Queen Square Institute of Neurology, University College London, London WC1N 3BG, UK; Department of Bioengineering, University of Pennsylvania, Philadelphia, PA 19104, USA; Department of Clinical and Experimental Epilepsy, UCL Queen Square Institute of Neurology, University College London, London WC1N 3BG, UK; Department of Brain Repair and Rehabilitation, UCL Queen Square Institute of Neurology, University College London, London WC1N 3BG, UK; Department of Clinical and Experimental Epilepsy, UCL Queen Square Institute of Neurology, University College London, London WC1N 3BG, UK; School of Biomedical Engineering and Imaging Sciences, King’s College London, London, UK; Victor Horsley Department of Neurosurgery, National Hospital for Neurology and Neurosurgery, London WC1N 3BG, UK; Department of Clinical and Experimental Epilepsy, UCL Queen Square Institute of Neurology, University College London, London WC1N 3BG, UK; Victor Horsley Department of Neurosurgery, National Hospital for Neurology and Neurosurgery, London WC1N 3BG, UK; Department of Clinical and Experimental Epilepsy, UCL Queen Square Institute of Neurology, University College London, London WC1N 3BG, UK; Department of Clinical and Experimental Epilepsy, UCL Queen Square Institute of Neurology, University College London, London WC1N 3BG, UK; Department of Clinical and Experimental Epilepsy, UCL Queen Square Institute of Neurology, University College London, London WC1N 3BG, UK; Department of Clinical and Experimental Epilepsy, UCL Queen Square Institute of Neurology, University College London, London WC1N 3BG, UK; Interdisciplinary Computing and Complex BioSystems Group, School of Computing, Newcastle University, Newcastle upon Tyne NE4 5TG, UK; Department of Clinical and Experimental Epilepsy, UCL Queen Square Institute of Neurology, University College London, London WC1N 3BG, UK; Department of Clinical and Experimental Epilepsy, UCL Queen Square Institute of Neurology, University College London, London WC1N 3BG, UK; Victor Horsley Department of Neurosurgery, National Hospital for Neurology and Neurosurgery, London WC1N 3BG, UK; Institute of Neuroscience, Cleveland Clinic London, London SW1X 7HY, UK; Department of Clinical and Experimental Epilepsy, UCL Queen Square Institute of Neurology, University College London, London WC1N 3BG, UK; Department of Clinical and Experimental Epilepsy, UCL Queen Square Institute of Neurology, University College London, London WC1N 3BG, UK; Department of Clinical and Experimental Epilepsy, UCL Queen Square Institute of Neurology, University College London, London WC1N 3BG, UK; Department of Clinical and Experimental Epilepsy, UCL Queen Square Institute of Neurology, University College London, London WC1N 3BG, UK; Victor Horsley Department of Neurosurgery, National Hospital for Neurology and Neurosurgery, London WC1N 3BG, UK; Institute of Neuroscience, Cleveland Clinic London, London SW1X 7HY, UK

**Keywords:** epilepsy, disconnection, thalamocortical, epilepsy surgery, frontal lobe

## Abstract

Around 50% of patients undergoing frontal lobe surgery for focal drug-resistant epilepsy become seizure free post-operatively; however, only about 30% of patients remain seizure free in the long-term. Early seizure recurrence is likely to be caused by partial resection of the epileptogenic lesion, whilst delayed seizure recurrence can occur even if the epileptogenic lesion has been completely excised. This suggests a coexistent epileptogenic network facilitating ictogenesis in close or distant dormant epileptic foci. As thalamic and striatal dysregulation can support epileptogenesis and disconnection of cortico-thalamostriatal pathways through hemispherotomy or neuromodulation can improve seizure outcome regardless of focality, we hypothesize that projections from the striatum and the thalamus to the cortex may contribute to this common epileptogenic network.

To this end, we retrospectively reviewed a series of 47 consecutive individuals who underwent surgery for drug-resistant frontal lobe epilepsy. We performed voxel-based and tractography disconnectome analyses to investigate shared patterns of disconnection associated with long-term seizure freedom. Seizure freedom after 3 and 5 years was independently associated with disconnection of the anterior thalamic radiation and anterior cortico-striatal projections. This was also confirmed in a subgroup of 29 patients with complete resections, suggesting these pathways may play a critical role in supporting the development of novel epileptic networks.

Our study indicates that network dysfunction in frontal lobe epilepsy may extend beyond the resection and putative epileptogenic zone. This may be critical in the pathogenesis of delayed seizure recurrence as thalamic and striatal networks may promote epileptogenesis and disconnection may underpin long-term seizure freedom.

## Teaser

About 50% of patients undergoing frontal lobe surgery for focal drug-resistant epilepsy become seizure-free post-operatively, but only about 30% remain seizure-free in the long-term. By retrospectively reviewing a series of operated individuals, Giampiccolo *et al.* show that cortico-thalamostriatal disconnection is linked to long-term seizure freedom.

**See Inati and Zaghloul (https://doi.org/10.1093/brain/awad141) for a scientific commentary on this article.**

## Introduction

Seizure outcome is variable in drug-resistant frontal lobe epilepsy, with seizure freedom at 1 year achieved in only 50% of patients,^1,2^ while long-term seizure freedom occurs in less than 30% of cases.^3^ Two different mechanisms have been proposed for early and delayed seizure recurrence: seizure recurrence within the first year is thought to be due to partial resection of the epileptogenic lesion or zone,^1^ whilst a 5–8% yearly risk of seizure recurrence within 5 years postoperatively^4^ may reflect ongoing epileptogenesis, despite complete resection of the primary abnormality.^4^ Animal models indicate seizures spread along long-range white matter pathways.^5^ Thus it is possible that seizure recurrence, despite complete resection of the epileptogenic lesion, may involve an epileptogenic network beyond the lesion whose dysfunction could be associated with seizure propagation and new or ongoing epileptogenesis.^6^ How this network is composed and if the pathways involved are shared amongst patients remains poorly understood.

Seizures can be modulated by lesioning or stimulating discrete brain networks.^7–9^ A role for thalamic networks in ictogenesis was demonstrated as early as the 1940s, as thalamic stimulation was shown to induce seizures^10^ and thalamocortical dysfunction was linked to seizure propagation,^11,12^ not only generalized seizures.^13^ Within these networks, connections specifically from the anterior thalamic nucleus and the centromedian nucleus to the cortex are relevant, as their virtual disconnection through neuromodulation can diminish or abolish seizures.^7,14–17^ Their role in resective surgery, however, has not been addressed. Cortico-cortical and cortico-striatal connections may also be involved in frontal seizure propagation, as surgeries performed with the aim to disconnect rather than resect, such as callosotomy^18^ or hemispherotomy,^19,20^ can decrease seizure frequency and severity independent of lesion location or focality. As common networks within the frontal lobe have been linked to seizure modulation, a unifying hypothesis could posit that individuals with frontal lobe epilepsy may share a common epileptogenic network which affects seizure outcome somewhat irrespective of lesion localization, which could potentially become a target for disconnection.

To test this hypothesis, we retrospectively reviewed 47 patients who underwent surgery for drug-resistant frontal lobe epilepsy and applied disconnectome analyses to highlight patterns of disconnection associated with long-term seizure freedom.

## Materials and methods

### Participants and seizure outcome

We performed a retrospective case collection, initially reviewing clinical data of 80 consecutive patients who underwent frontal lobe epilepsy surgery at our institution from 2007 to 2021. Of these, we identified 47 patients who met the following criteria: (i) preoperative and three-month postoperative MRI; and (ii) seizure outcome reported after at least 3 years. We evaluated lesion side, epilepsy duration, history of focal-to-bilateral tonic-clonic seizures, histology, seizure outcome and resection volume, and classified seizure outcome as (i) seizure-free (International League against Epilepsy grade I,^21^ ILAE 1); or (ii) continuing seizures (ILAE 2 or higher). In those cases in whom (i) there was no visible residual lesion on postoperative MRI and (ii) histology confirmed a lesion/malformation, resection was considered complete. The study was conducted in accordance with ethical standards of the Declaration of Helsinki and was approved by the Research Ethics Committee of our institution (IRB: 22/SC/0016).

### Presurgical evaluation

A workflow of presurgical evaluations at our Institution has been detailed as in Duncan^22^ and descriptive surgical cases can be found in the Supplementary material. Initially patients were assessed with history of epilepsy, drug resistance, comorbidities, and had volumetric MRI, video-EEG telemetry, neuropsychiatric and neuropsychological assessments. Handedness was determined using a standardized questionnaire.^23^ Language lateralization was determined using language functional MRI (fMRI)^24^ or Wada test. Magnetoencephalography (MEG), EEG-fMRI, ictal single photon emission CT (SPECT) and fluorodeoxyglucose (FDG)-PET were carried out as indicated. These data were discussed by a multidisciplinary team of neurologists, neurophysiologists, neurosurgeons, neuropsychologists and neuropsychiatrists.^22^ For individuals in whom results from semiology, imaging, neuropsychology and electrophysiology were not definitive, invasive EEG recordings were performed to localize the epileptogenic zone. Stereoencephalography (SEEG), surface electrodes (grid or strip electrodes) or combined depth and surface electrodes were tailored to individual patients.

### Imaging acquisition and processing

Structural MRI datasets were collected preoperatively and at a minimum follow-up of 3 months on a 1.5 T or a 3 T MR imaging scanner (General Electric). Three-month postoperative T_1_ MRIs were used to exclude immediate postoperative brain swelling and brain-shift.

MRI sequences used for evaluation included standard 3D isovolumetric (1 mm) T_1_-weighted sequence with inversion-recovery fast spoiled gradient recalled echo [echo time (TE) 3.1 ms, repetition time (TR) = 7.4 ms, inversion time = 400 ms, field of view (FOV) = 224 × 256 × 256 mm, matrix = 224 × 256 × 256, voxel size = 1.00 × 1.00 × 1.00 mm = 1.00 mm^3^, parallel imaging acceleration factor = 2] and a coronal dual-echo fast recovery fast spin echo proton-density/T_2_-weighted sequence (TE = 30/119 ms, TR = 7600 ms, FOV = 220 × 220 mm, matrix = 512 × 512, slice thickness = 4 mm, voxel size = 0.43 × 0.43 × 4.00 mm = 0.74 mm^3^, SENSE factor = 2). Each individual resection cavity was manually drawn from the postoperative MRI using ITKsnap (http://www.itksnap.org).^25^ The individual brain anatomy with the related resection cavity was normalized to Montreal Neurological Institute (MNI)-152 standard space using an enantiomorphic normalization approach using SPM12 (https://www.fil.ion.ucl.ac.uk/spm/software/spm12/).^26^

Diffusion MRI consisted of (i) a single-shell acquisition using a cardiac-triggered single-shot spin-echo planar imaging sequence (1.875×1.875×2.4 mm resolution, 52 directions, 6 b0, b-value:1200 s/mm^2^); and (ii) a multi-shell acquisition (1.6 mm isotropic resolution, 101 directions, 14 b0, b-values: 300, 700 and 2500 s/mm^2^). Diffusion MRI data were corrected for noise, Gibbs ringing and signal drift using MRtrix 3 (https://www.mrtrix.org).^27^ Distortion correction was performed using a synthesized b0 (Synb0-DisCo) produced from a T_1_-weighted MRI.^28^ The result was then included into FSL's Topup. Magnetic susceptibility field, eddy current and motion artifact correction were performed using FSL (https://fsl.fmrib.ox.ac.uk/fsl).^29^ Response functions for CSF and white and grey matter were estimated using Single-Shell 3-Tissue Constrained spherical deconvolution (CSD) and Multi-Shell 3-Tissue CSD in MRtrix 3. Single- and multi-shell whole brain probabilistic high angular resolution diffusion imaging was computed using StarTrack (https://www.mr-startrack.com/), selecting individual grey and white matter regions as masks. Spherical deconvolution was based on a modified damped Richardson–Lucy algorithm^30^ with the following parameters: fibre response *α* = 1.5; number of iterations = 300; amplitude threshold *η* = 0.0015; geometric regularization *ν* = 16. Fibre tracking was performed according to the following parameters: minimum Hindrance Modulated Orientational Anisotropy (HMOA) threshold = 0.001; number of seeds per voxel = 10; maximum angle threshold = 45°; minimum fibre length = 20 mm; maximum fibre length = 300 mm.^26^

### Tractwise and atlas-based disconnectome analysis

First, we computed the disconnectome map of each patient using the BCBtoolkit (http://www.bcblab.com). This map shows, for each voxel, the probability of having disconnected white matter fibres passing through it, based on a healthy population tractography dataset resulting in the associated disconnectome maps (tractwise voxel-based analysis).^31,32^ This is achieved by computing a tractogram of fibres passing through the cavity in each of 20 unrelated healthy right-handed adults from the 7 T dataset of the Human Connectome Project, processed using High Angular Resolution Diffusion Imaging tractography (spherical deconvolution). For each patient lesion, 20 tractograms are generated and overlayed to produce a probabilistic map that takes inter-individual variability into account. A sample of 20 was chosen as this accounts for more than 80% of shared variance in the overall population.^31^ Specific tract disconnection was then assessed using white matter atlases^33,34^ (atlas-based analysis) in Tractotron.

For disconnectome maps and tract disconnection from each cavity, a probability of disconnection above the level of chance was selected, as previously.^32^ Each patient's disconnection profile was used to investigate if disruption of specific white matter pathways were associated with seizure outcome. In the tractwise voxel-based analysis, non-parametric two sample *t*-tests were performed on disconnection maps using FSL's Randomise with 5000 permutations, family-wise error-correction and threshold-free cluster enhancement^35^ to determine which disconnection profiles were associated with seizure outcome at the different time-points. In the atlas-based analysis, a chi-square analysis with Bonferroni correction was performed using tract disconnection (disconnected versus non-disconnected) in Tractotron and seizure outcome (seizure-free versus seizure-relapsing) according to the side of resection (left-sided tracts were evaluated in left-sided resections and right-sided tracts in right hemisphere resection). A flow-chart describing the disconnectome analysis can be found in Supplementary Fig. 1 and in previous reports.^36,37^

### Tractography-based disconnectome analysis

Preoperative tractography was acquired in seventeen of the operated patients. Tracts were selected for dissection according to the results of the previous disconnectome analysis, with dissection performed in each patient's DWI native space.^38^ Preoperative MRI, postoperative MRI and the resection cavity were coregistered to the preoperative diffusion data using the Elastix registration toolbox (https://github.com/lassoan/SlicerElastix) in 3D Slicer. Virtual dissection for white matter pathways was performed manually as detailed in Rojkova and colleagues^33^ using a constrained region of interest-based approach. The number of streamlines that were intersecting with the resection cavity was used to calculate a percentage of disconnection for each relevant tract. An independent *t*-test was performed to test percentage of disconnection associated with seizure outcome.

A secondary tractography analysis was performed on the same normative dataset of 20 healthy subjects. After relevant white matter tracts were dissected in each subject, percentages of disconnection were calculated with the patients’ resection cavities normalized in MNI space. Percentages of tract disconnection were then averaged according to each of the 47 resection cavities and independent *t*-test was performed to test whether percentage of disconnection was associated with seizure outcome.

### Neuropsychological assessment

Expressive language (Graded Naming Test),^39^ executive function (phonemic fluency and semantic fluency)^40,41^ and memory function (BIRT Memory and Information Processing Battery (BMIPB)^42^ were assessed preoperatively and 12 months after surgery. The Graded Naming Test consists of oral naming of 30 black and white pictures.^39^ In phonemic and semantic fluency^40,41^ tasks, the subject produces as many words as possible in 1 min according to category (phonemic fluency: words starting with the letter ‘S’; semantic fluency: ‘animals’). In the memory task, participants are read a list of 15 words and then are asked to recall them. The task comprises five trials with performance measured by the sum of recalled words.^42^ To evaluate neuropsychological outcome we used reliable change indices (RCIs) using a 90% confidence interval^43–45^ and grouping performance binarily (deteriorated or not).

**Table 1:** Patient demographics

Pt.	Sex	Age (years)	Epilepsy duration (years)	Operated hemisphere	Resection cavity (mm^3^)	EoR	Presumed cortical localization	FBTCS	Histology	Seizure outcome	
										3 y	5 y
1	M	29	15	Left	4998	T	SFG	Yes	DNT	SR	SR
2	M	33	21	Left	5284	ST	MFG	Yes	GL (II)	SF	–
3	M	29	20	Left	1832	T	M1	Yes	DNT	SF	–
4	M	52	50	Left	61 289	T	MFG	Yes	FCD	SR	SR
5	F	27	25	Left	12 762	T	pTRI	No	FCD IIB	SR	SR
6	M	34	22	Left	68 011	ST	SFG	No	Gliosis	SF	SR
7	F	17	3	Left	6346	ST	pOP, Insula	No	GL (II)	SF	SF
8	F	24	17	Left	17 045	T	SMA	Yes	FCD IIB	SF	SF
9	F	49	47	Left	43 082	T	SFG	Yes	FCD IIB	SF	–
10	M	37	32	Left	6069	T	SFG, MFG	Yes	FCD IIB	SF	SF
11	F	17	7	Left	37 566	ST	pOP, pTRI	Yes	DNT	SR	SR
12	M	23	12	Left	70 502	T	OFC	Yes	FCD IIA	SR	SR
13	F	39	18	Left	7248	T	pTRI, pOP	Yes	DNT	SF	SF
14	M	29	17	Left	14 615	T	SFG	Yes	FCD IIB	SF	–
15	F	53	23	Left	44 341	T	OFC	Yes	DNT	SR	SR
16	F	36	14	Left	16 777	ST	SMA	No	NAD	SR	SR
17	M	28	12	Left	20 262	ST	pTRI, OFC	No	GL (I)	SR	SR
18	M	17	16	Left	33 747	ST	pTRI, OFC	Yes	NAD	SR	SR
19	F	27	26	Left	56 862	T	SFG	Yes	FCD IIA	SF	SF
20	F	60	34	Left	7859	T	MFG	No	FCD IIB	SR	–
21	M	56	48	Left	7293	T	MFG, M1	No	FCD IIB	SF	SF
22	F	37	31	Left	19 327	T	SFG	No	FCD IIB	SR	–
23	F	42	35	Left	14 753	T	SMA	Yes	FCD IIB	SR	–
24	M	30	17	Left	36 461	ST	SFG	Yes	NAD	SR	SR
25	F	42	4	Left	4321	ST	OFC	No	CAV	SF	–
26	F	35	4	Left	3294	T	pOP, M1	No	CAV	SF	–
27	F	35	15	Right	104 092	ST	OFC, SFG	No	Gliosis	SR	SR
28	M	23	8	Right	4867	T	ORB	No	DNT	SF	SF
29	M	45	42	Right	8008	T	SMA	No	FCD IIB	SF	SF
30	M	45	40	Right	5042	ST	pTRI, pOP	Yes	Gliosis	SR	SR
31	M	40	15	Right	53 593	T	SFG	Yes	FCD IIB	SF	SF
32	F	59	42	Right	26 708	T	MFG, pTRI	No	FCD IIB	SR	SR
33	F	30	28	Right	76 685	T	pOP, pTRI	Yes	FCD IIB	SF	SF
34	F	28	18	Right	22 963	T	SMA	Yes	FCD IIB	SF	SF
35	F	28	11	Right	4203	T	pTRI, pOP	Yes	CAV	SR	–
36	M	26	11	Right	77 866	T	OFC, pORB	No	FCD	SF	–
37	M	39	33	Right	13 425	ST	SFG, MFG	Yes	Gliosis	SR	SR
38	F	32	19	Right	22 774	ST	SMA	Yes	NAD	SR	–
39	M	32	9	Right	42 817	ST	OFC, pORB	No	Gliosis	SF	SF
40	F	37	30	Right	2833	T	AntCING	Yes	DNT	SF	–
41	M	47	39	Right	58 710	ST	OFC, SFG	Yes	Gliosis	SF	SR
42	F	38	–	Right	15 540	ST	SFG, OFC	No	Gliosis	SR	SR
43	F	29	22	Left	24 346	ST	pOP, pTRI, MFG	Yes	DNT	SF	SF
44	M	46	39	Right	45 288	T	pOP, Insula	No	FCD IIB	SF	SF
45	M	35	27	Right	87 550	ST	SMA	No	Gliosis	SF	SR
46	F	22	16	Left	62 349	T	SFG	Yes	FCD	SR	–
47	F	47	34	Left	52 114	T	MFG, SFG	Yes	FCD IIB	SR	SR

antCING = anterior cingulate gyrus; EoR = extent of resection; F = female; FCD = focal cortical dysplasia; FBTCS = focal to bilateral tonic clonic seizure; GL = glioma; M = male; MFG = middle frontal gyrus; M1 = precentral gyrus; NAD = no appreciable disease; OFC = orbitofrontal cortex; Pt. = patient; pOP = pars opercularis; pTRI = pars triangularis; pORB = pars orbitalis; SFG = superior frontal gyrus; SMA = supplementary motor area; ST = subtotal; SF = seizure free; SR = seizure recurrence; T = total.

### Statistical analysis

Statistical analysis was performed using SPSS Statistics (28, IBM, Armonk, NY, USA). Normality of variables distribution were evaluated using a Kolmogorov–Smirnov test. A chi-square analysis with Bonferroni correction was used to evaluate the association between seizure outcome and histology or focal-to-bilateral tonic-clonic seizures in the different time-points. An independent two sample *t-*test was used to test whether resection volumes were associated seizure outcome. An atlas-based chi-square analysis with Bonferroni correction was used to evaluate tracts involved in seizure outcome or neuropsychological outcome at the different time-points. Non-parametric voxel-based two-sample *t-*tests were performed with FSL’s Randomise using threshold-free cluster enhancement and corrected for family-wise error rate^35^ to evaluate each voxel involved in seizure outcome at the different time-points. Independent two sample *t-*tests were used to assess how percentage of tract disconnection was associated with seizure outcome or neuropsychological outcome.

### Data availability

Anonymized data that support the findings of this study are available from the corresponding authors upon reasonable request.

## Results

### Participants and seizure outcome

**Figure 1:**
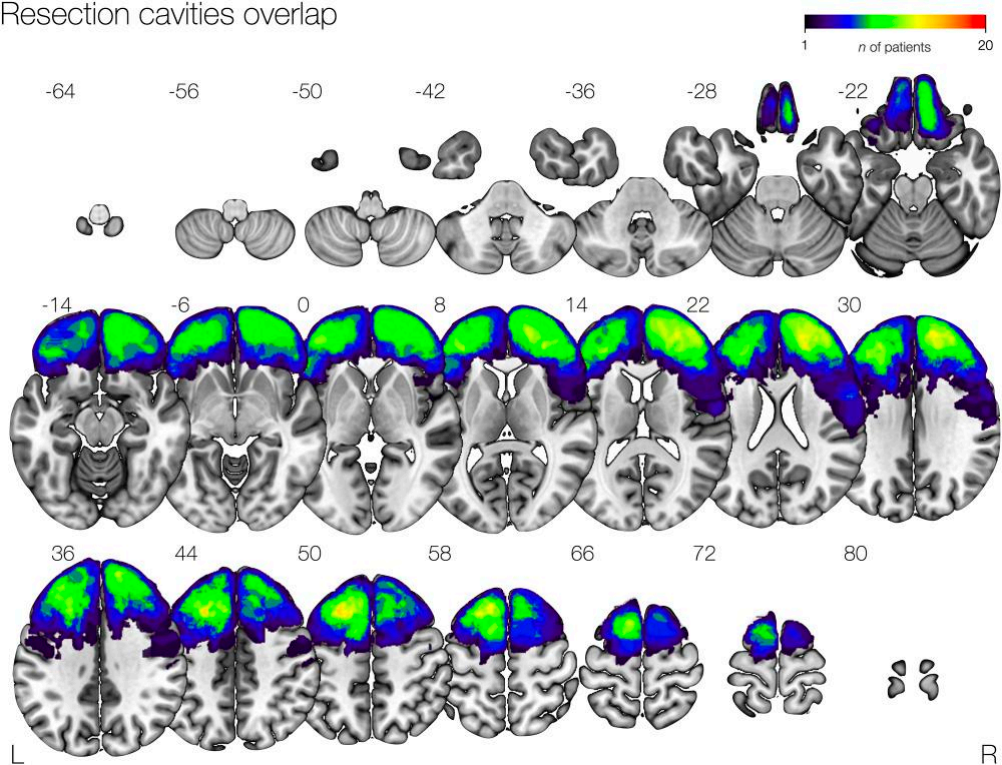
**Overlap map of resection cavities in the patients’ cohort.** A map of all resection cavities normalized to the MNI space is shown, with a colour bar showing voxel overlapping in a maximum of 20 patients.

We identified 47 frontal lobe epilepsy patients that underwent surgical resection of the epileptic focus (29 left hemisphere, 18 lesional). Demographics are shown in Table 1 and the overlap of all 47 resection cavities is shown in Fig. 1. The mean duration of epilepsy before surgery was 23 (±12; between 3–50) years. The most common histology findings were cortical dysplasia (21 patients) followed by dysembryoplastic neuroepithelial tumour (DNT; *n* = 8), cavernoma (*n* = 3) and glioma (*n* = 3). In the remaining 12 patients, histopathology was not diagnostic. Resection was considered complete in 29 cases. Of 47 patients, 25 were seizure free (ILAE 1) at 3 years. Of 33 patients followed-up for 5 years, 14 patients remained seizure free. The median resection volume was 20.2 cc (IQR: 52-7 cc). Histology was not associated with seizure outcome at 3 or 5 years. There was no relationship between history of focal-to-bilateral tonic-clonic seizures and seizure outcome at any time point. There was no correlation between resection volume and seizure outcome [3 years: *t*(45) = 0.171; *P* = 0.865; 5 years: *t*(31) = 1.431; *P* = 0.162], nor between epilepsy duration and seizure outcome [3 years: *t*(44) = 0.314; *P* = 0.755; 5 years: *t*(30) = 0.305; *P* = 0.762] in our patient cohort. Partial resection was not associated with a worse seizure outcome 3 years after surgery [χ^2^(1) = 0.896, *P*_corrected_ = 0.344]; however, it was significantly associated with seizure recurrence after 5 years [χ^2^(1) = 9.916, *P*_corrected_ < 0.017].

**Table 2:** Patient presurgical invasive and non-invasive investigations

Pt.	Handedness	fMRI	Tractography	Invasive recordings	MEG	PET-FDG	Ictal SPECT	fMRI-EEG
1	R	Yes	No	Combined	Yes	No	No	No
2	R	Yes	Yes	No	No	No	No	No
3	R	Yes	Yes	No	No	Yes	No	No
4	R	No	No	Combined	No	No	No	No
5	L	No	No	Combined	No	No	No	No
6	R	No	No	Combined	No	No	No	No
7	R	No	No	No	No	No	No	No
8	R	Yes	No	Combined	Yes	Yes	Yes	Yes
9	L	Yes	Yes	Combined	Yes	Yes	Yes	No
10	R	Yes	Yes	No	No	Yes	Yes	No
11	R	Yes	Yes	Combined	Yes	Yes	Yes	No
12	R	Yes	No	Surface	Yes	Yes	Yes	Yes
13	R	Yes	No	No	No	No	No	No
14	R	Yes	Yes	Combined	No	No	Yes	Yes
15	R	Yes	No	SEEG	Yes	Yes	Yes	Yes
16	R	Yes	No	Surface	No	Yes	No	No
17	R	Yes	No	Surface	Yes	Yes	Yes	Yes
18	R	Yes	No	Surface	Yes	Yes	Yes	Yes
19	L	Yes	Yes	Surface	Yes	Yes	Yes	No
20	L	Yes	Yes	Combined	No	No	No	No
21	L	Yes	No	Combined	No	No	No	No
22	R	Yes	No	Combined	Yes	Yes	No	No
23	L	Yes	Yes	No	No	Yes	Yes	No
24	L	Yes	No	SEEG	Yes	Yes	No	No
25	R	Yes	Yes	No	No	No	No	No
26	R	Yes	No	No	No	Yes	No	No
27	R	Yes	No	SEEG	No	No	No	No
28	R	Yes	No	No	No	No	No	No
29	R	Yes	No	Surface	No	No	No	No
30	R	Yes	No	Combined	Yes	No	No	Yes
31	R	Yes	No	Combined	Yes	Yes	No	Yes
32	R	Yes	No	Combined	No	Yes	Yes	No
33	R	Yes	Yes	SEEG	No	Yes	No	No
34	R	Yes	Yes	Surface	No	Yes	Yes	No
35	R	Yes	Yes	No	No	No	No	No
36	R	Yes	Yes	SEEG	Yes	Yes	No	No
37	R	Yes	No	Combined	Yes	No	No	No
38	R	Yes	Yes	SEEG	No	Yes	No	No
39	R	Yes	No	SEEG	Yes	Yes	No	No
40	R	Yes	No	No	Yes	Yes	Yes	No
41	R	Yes	Yes	SEEG	Yes	Yes	No	No
42	R	No	No	No	No	No	No	No
43	R	No	No	Surface	No	No	No	No
44	R	Yes	No	No	No	No	No	No
45	R	Yes	No	SEEG	No	Yes	No	No
46	R	Yes	No	Surface	Yes	Yes	No	No
47	R	Yes	Yes	SEEG	Yes	Yes	No	No

Combined = combined surface and depth electrodes; L = left; Pt. = patient; R = right; Surface = grid or strip electrodes.

### Presurgical evaluation

Data on presurgical non-invasive and invasive investigations are summarized in Table 2. Example surgical cases are included in the Supplementary material. In total, 13/47 (27%) of patients underwent resection without invasive recordings. Of these, 11 underwent fMRI, 5 FDG-PET, 3 ictal SPECT and 1 MEG. Thirty-four (73%) underwent invasive recording (10 patients had SEEG, 9 had surface electrodes in the form of grid or strip electrodes and 15 had combined surface and depth electrodes). Of these, 30 underwent fMRI, 22 FDG-PET, 19 MEG, 11 ictal SPECT and 8 EEG-fMRI. The most commonly resected regions are highlighted in Fig. 1.

**Table 3:** Patients’ neuropsychological evaluation

Graded Naming Task	Phonemic fluency	Semantic fluency	BMIBP
Preoperative			
ȃ*n* = 47	*n* = 46	*n* = 46	*n* = 45
ȃ17 ± 5	12.4 ± 6.1	17.5 ± 7.1	46.7 ± 10.3
1-year postoperative			
ȃ*n* = 40	*n* = 40	*n* = 41	*n* = 38
ȃ18.4 ± 5.1	11.6 ± 6.7	17.4 ± 6.3	45.3 ± 11.8

Averaged number of correct item per category is shown.

GNT = Graded Naming Test; BMIPB = Brain Injury Rehabilitation Trust (BIRT) Memory and Information Processing Battery; *n* = number of patients.

### Disconnectome analysis

#### Tractwise voxel-based disconnectome analysis

**Figure 2:**
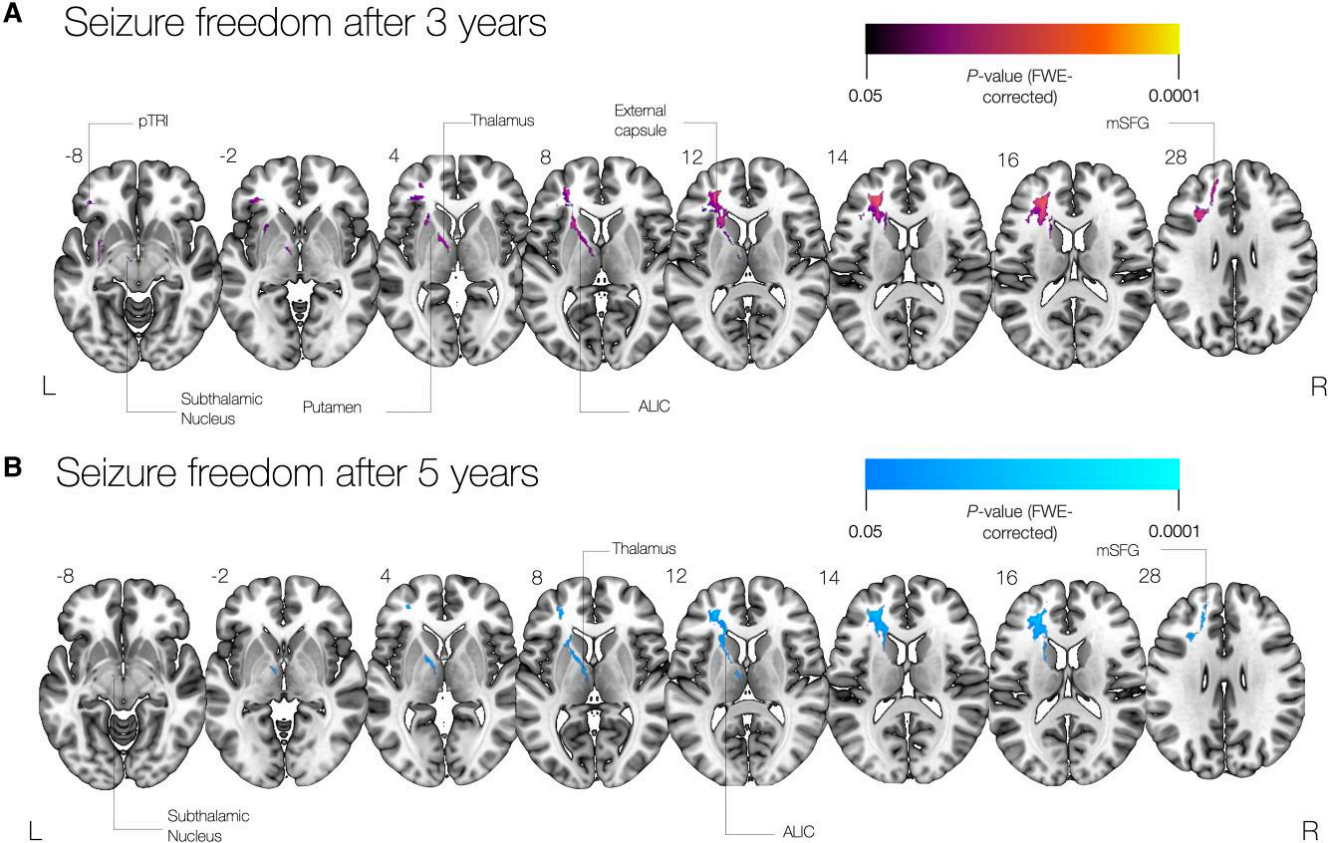
**Tractwise voxel-based analysis of disconnectome maps**. (**A**) A non-parametric two sample *t*-test shows that disconnection of thalamic, putaminal and subthalamic regions as well as in the anterior prefrontal cortex is associated with a better seizure outcome 3 years after surgery. (**B**) A non-parametric two sample *t*-test shows that disconnection of thalamic, putaminal and subthalamic fibres projecting to the anterior prefrontal cortex is associated with a better seizure outcome 5 years after surgery. Cortical terminations: ALIC = anterior limb of the internal capsule; pTRI = pars triangularis; mSFG = middle superior frontal gyrus.

A non-parametric two-sample *t*-test showed that after 3 years, seizure freedom was associated with disconnection of fibres involving the left anterior limb of the internal capsule, including the anterior thalamus, mediodorsal thalamus and striatum, the subthalamic nucleus and the dorsomesial, dorsolateral and ventrolateral prefrontal cortices (Fig. 2A). A similar result was also present at 5 years (Fig. 2B).

#### Atlas-based disconnectome analysis

**Figure 3:**
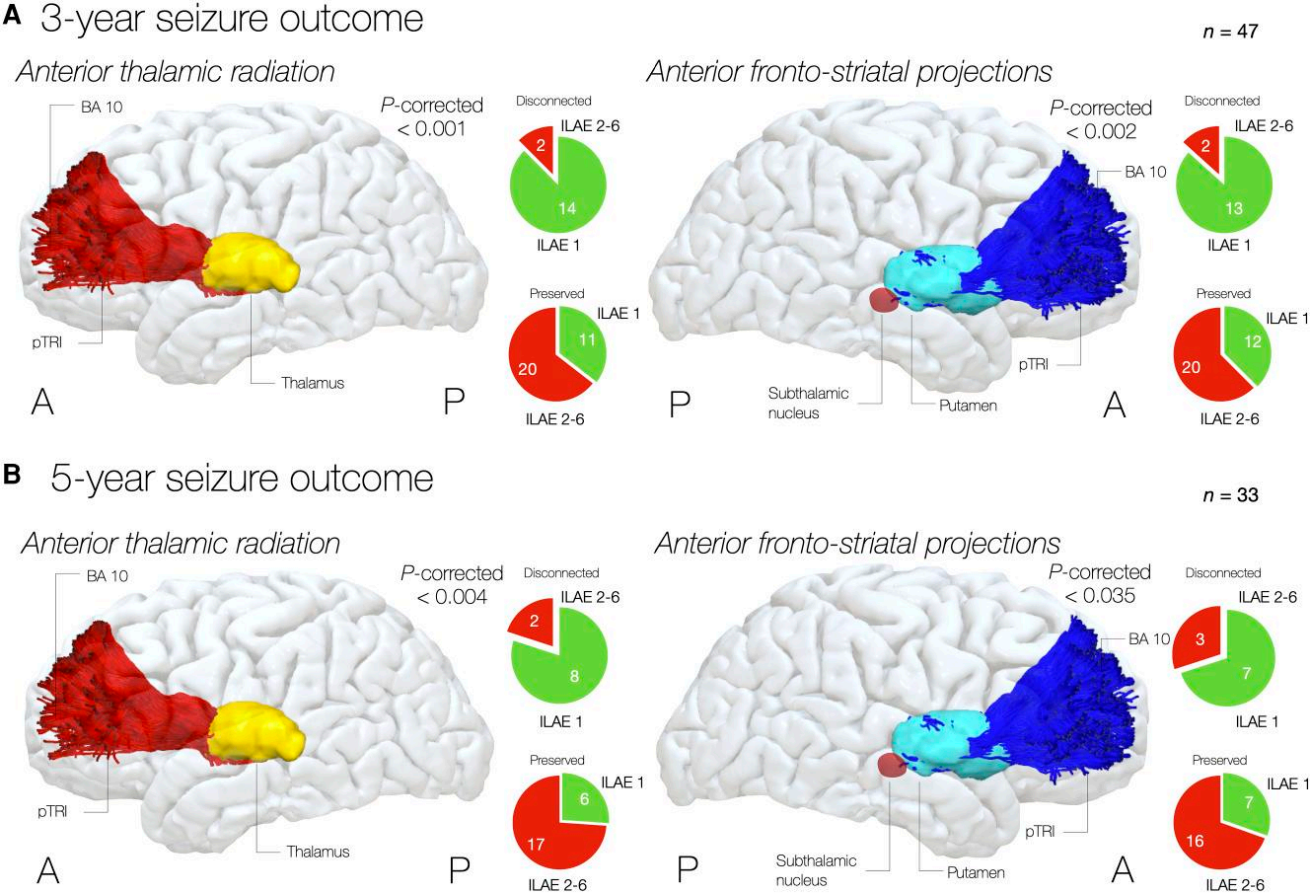
**Atlas-based disconnectome analysis.** (**A**) After 3 years, disconnection of anterior thalamic and striatal projections was associated with seizure freedom. Pie charts show patients being seizure free (green, ILAE 1) or seizure-relapsing (red, ILAE 2–6) according to each tract being disconnected or preserved. Each tract group includes the same tract in the left and right hemisphere. (**B**) Also, after 5 years, disconnection of anterior thalamic and striatal projections was associated with seizure freedom. Pie charts show patients being seizure free (green, ILAE 1) or seizure-relapsing (red, ILAE 2–6) according to each tract being disconnected or preserved. As for above, each tract group includes the same tract in the left and right hemisphere. Subcortical structures: red = anterior thalamic radiation; blue = fronto-striatal; light yellow = thalamus; light blue = putamen; dark red = subthalamic nucleus. Cortical terminations: BA 10 = Brodmann area 10; mSFG = middle superior frontal gyrus; pTRI = pars triangularis.

The previous analysis suggested subcortical locations are involved in seizure freedom but was unable to distinguish specific white matter tracts. We therefore conducted an atlas-based analysis (see Supplementary Table 1 for disconnectome data of significant pathways) to identify which fibre tracts underpin the previous tractwise voxel-based analysis. This highlighted that disconnection of anterior cortico-thalamostriatal projections was associated with better seizure outcome after 3 years: only 2/16 (12%) patients with the anterior thalamic radiation disconnected had seizure recurrence, compared with 20/31 (64%) in whom the anterior thalamic radiation was preserved [χ^2^(1) = 11.468, *P*_corrected_ < 0.001, respectively]. Similarly, only 2/15 (13%) of patients with anterior corticostriatal disconnection had seizure recurrence, compared with 20/32 (63%) in whom cortico-striatal pathways were preserved [χ^2^(1) = 9.916, *P*_corrected_ < 0.002]. Preservation of these tracts was also linked with seizure freedom at 5 years post-surgery: seizure recurrence was observed in only 2/10 (20%) patients after disconnection of the anterior thalamic radiation [χ^2^(1) = 8.294, *P*_corrected_ < 0.004] compared with 17/23 (74%) of those in whom these tracts were preserved, and in only 3/10 (30%) patients after anterior corticostriatal disconnection [χ^2^(1) = 4.467, *P*_corrected_ < 0.035], compared with 16/23 (70%). (Fig. 3).

To rule out whether worse long-term seizure outcome was caused by partial resection, we performed a second analysis to test the influence of cortico-thalamostriatal disconnection on long-term seizure outcome in those 29 patients for whom total resection of the lesion was achieved (18/29 patients at the 5-year follow-up). At the 3-year follow-up, only one of the 12 patients in whom the anterior thalamocortical projection was resected had seizure recurrence compared to 11/17 patients (65%) for whom this was preserved [χ^2^(1) = 9.216, *P*_corrected_ < 0.002]. Similarly, none of the 10 patients with anterior cortico-striatal disconnection had seizure recurrence as compared to 12/19 patients (63%) in whom this was preserved [χ^2^(1) = 10.774, *P*_corrected_ < 0.001]. There were similar findings at the 5-year follow-up: none of the seven patients with disconnection of the anterior thalamocortical radiation had seizure recurrence, compared with 7/11 (64%) in whom this was preserved [χ^2^(1) = 8.061, *P*_corrected_ < 0.007] and none of the six patients with anterior cortico-striatal disconnection had seizure recurrence, compared with 7/12 (58%) patients in whom this was preserved [χ^2^(1) = 5.727, *P*_corrected_ < 0.017]. There was no association between cortico-thalamostriatal disconnection and seizure outcome at the different time-points in the 11 patients with partial resections.

#### Tractography-based disconnectome analysis

First, we performed tractography dissections in a subgroup of 17 patients who also underwent preoperative tractography to examine patterns of disconnection linked to patient-specific anatomy. Results from the tractography analysis confirmed those from the voxel-based and atlas-based analyses: at three years, patients with anterior thalamic disconnection showed a trend towards better seizure outcomes (11/17 patients were seizure free) with an average of 41% of streamlines resected in the seizure-free group versus 16% resected in those with seizure recurrence [*t*(15) = 2.08; *P* < 0.056], which was significant if the five patients with partial resections were excluded [8/12 patients seizure free; 41% versus 10% streamlines resected in seizure-free/seizure-recurrence groups, *t*(10) = 2.44; *P* < 0.035]. Only five patients had tractography at the 5-year follow-up, precluding statistical analysis.

**Figure 4:**
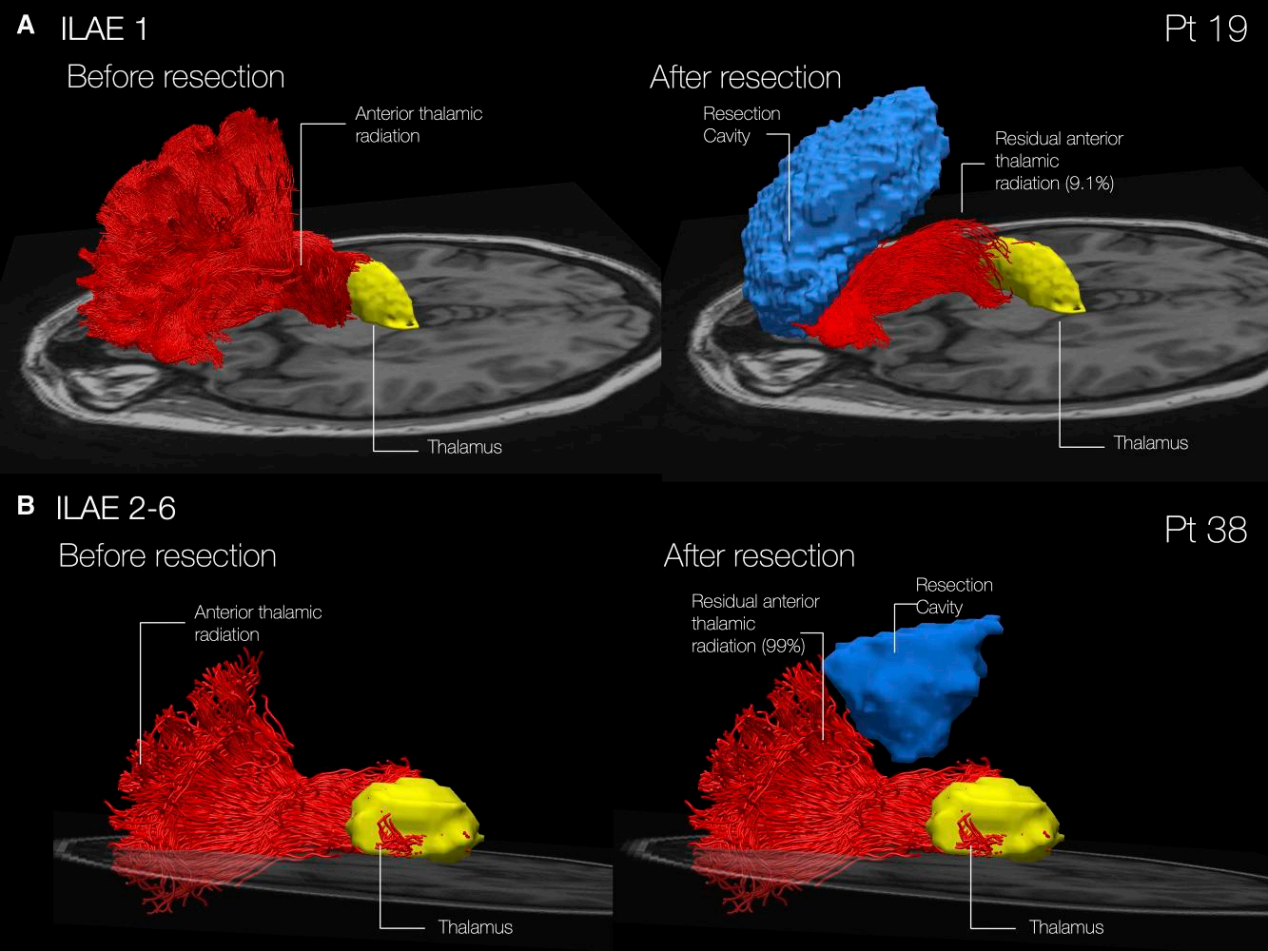
**Tractography-based disconnectome analysis.** (**A**) Disconnection in Patient 19 is shown. The patient remained seizure free 3 years after surgery. (**B**) Disconnection in Patient 38 is shown. After 3 years the patient had relapsing seizures. Red = anterior thalamic radiation; yellow = thalamus; light blue = resection.

We next ran a similar tractography analysis using normative data. This also showed disconnection of the anterior thalamic radiation was significantly associated with seizure outcome after 3 years [85% versus 64% streamlines resected in seizure-free/seizure-recurrence groups; *t*(45) = 3.125; *P* < 0.003] and after 5 years [87% versus 61% streamlines resected in seizure-free/seizure-recurrence groups; *t*(31) = 3.142; *P* < 0.004]. Disconnection of anterior corticostriatal projections was significantly associated with seizure freedom at the 3 year follow-up [74% versus 54% streamlines resected in seizure-free/seizure-recurrence groups; *t*(45) = 2.488; *P* < 0.017], and there was a trend towards a better seizure outcome at 5 years, although this did not reach significance [72% versus 55% streamlines resected in seizure-free/seizure-recurrence groups; *t*(31) = 1.684; *P* = 0.102]. Notably, disconnection of the anterior thalamic radiation was also associated with seizure freedom when selecting only patients who underwent complete resections [after 3 years: 88% versus 66% streamlines resected in seizure-free/seizure-recurrence groups, *t*(27) = 3.470; *P* < 0.002; after 5 years: 88% versus 64% streamlines resected in seizure-free/seizure-recurrence groups, *t*(16) = 2.846; *P* < 0.012], with disconnection of anterior corticostriatal projections being significantly associated with seizure freedom at 3 years [76% versus 54% streamlines resected in seizure-free/seizure-recurrence groups, *t*(27) = 2.114; *P* < 0.044] but only showing a trend towards a better seizure outcome at 5 years [69% versus 55% streamlines resected in seizure-free/seizure-recurrence groups, *t*(16) = 0.971; *P* = 0.346]. Importantly, there was no significant association between cortico-thalamostriatal disconnection and seizure outcome in patients with partial resections. As there was no significant difference in the amount of cortico-thalamostriatal streamlines disconnected between complete versus partial resections, these analyses may potentially suggest that disconnection supporting a better seizure outcome may not arise from just disconnection *per se* but may work combined with complete resections. Patterns of disconnection associated with long-term seizure outcome are displayed in Fig. 4, and data with disconnection in the individual patient and normative data are provided in Supplementary Tables 2 and 3.

#### Neuropsychological assessment

Neuropsychological data of our cohort of patients can be found in Table 3. We investigated whether disconnection of anterior thalamic and cortico-striatal projections was associated with a poorer neuropsychological outcome in patients tested for language, executive and memory function using atlas- and tractography-based disconnectome analyses. There was no significant association between disconnection of these pathways and a worse neuropsychological outcome in language, executive (phonemic and semantic fluency) and memory one year after surgery (Supplementary material).

## Discussion

In models of focal drug-resistant epilepsy, the epileptogenic zone is envisioned to be a localized cortical region and surgery aims for complete removal of this region which should lead to seizure freedom.^46^ However, data from neuromodulation studies in animals and humans,^9,14,17^ electrographic patterns of seizure spread^5^ and evidence from disconnective surgery^18,20,47^ all indicate a wider epileptogenic network exists whereby white matter connections may facilitate seizures.^6,48^ In the present study, we analysed retrospective data from 47 patients operated for frontal lobe epilepsy to investigate common patterns of disconnection that may be relevant for long-term seizure freedom. Our disconnectome analyses indicated that disconnection of anterior thalamic and corticostriatal projections is significantly associated with seizure freedom at 3 and 5 years, suggesting these white matter pathways may play a role in preventing delayed seizure recurrence. These findings indicate that cortico-thalamostriatal disconnection may be linked to long-term seizure freedom and are in agreement with previous evidence from animal^49^ and stimulation studies,^50^ which suggests that these fibres may modulate epileptogenesis. Our findings offer a novel framework to understand delayed seizure recurrence in focal frontal lobe epilepsy with direct implications for resective epilepsy surgery and long-term seizure freedom.

### Long-term seizure freedom and disconnection of anterior cortico-thalamostriatal projection

Late seizure recurrence occurs frequently after epilepsy surgery.^1,4^ This occurs predominantly within the first 5 years after surgery and is reportedly unrelated to the completeness of the lesion resection,^1^ compared with early seizure recurrence within the first 6 months, which is linked to partial lesion resection.^1,4^ This suggests structures outside the epileptogenic zone may have a role in supporting novel seizure generation. This hypothesis may be supported by our results which indicate that disconnecting anterior thalamocortical and anterior corticostriatal projections may contribute to prevent seizure recurrence after 3 and 5 years. A role for thalamocortical connections in supporting seizure spread has long been established,^10^ and thalamocortical dysregulation has been shown to be critical in focal-to-bilateral tonic-clonic seizures,^11^ absence seizures^51,52^ and other seizure types in genetic generalized epilepsies.^14,53,54^ Extra-thalamic projections have been proposed to impact seizure severity, with the basal ganglia—including the subthalamic nucleus^8^—possibly involved in modulating seizure activity.^8,9^ Stimulation of the anterior thalamic nucleus (ANT) in generalized and focal epilepsy may decrease or abolish seizures.^16,17^ Stimulation of diffuse thalamocortical projections through the centromedian nucleus (CM) modulates cortical activity^15^ and may decrease seizure frequency and severity.^14^ Projections within the anterior thalamic radiation may overlap with thalamic projections of the ANT, the CM and the medio-dorsal nucleus of the thalamus, as well as with striatal projections from the subthalamic nucleus, putamen and caudate to the orbitofrontal, dorsolateral and ventrolateral prefrontal cortex.^6,55,56^ Thus, it is tempting to speculate that disconnection of anterior thalamic and striatal projections may affect the same networks modulated in deep brain stimulation. Modulatory networks arising from the thalamus and other subcortical regions may thus play a role not only in seizure severity and maintenance of epileptic activity,^6,14,17^ but also in delayed seizure recurrence.

### Surgical relevance

Our results suggest that complete resection of the epileptogenic zone alone may be insufficient if coexisting networks involved in seizure generation and propagation are not disconnected. This is relevant for future developments in epilepsy surgery, as it suggests that tailored disconnection together with complete resection may increase chances of long-term seizure freedom. It is important to note that disconnection alone was not associated with long-term seizure freedom. This may suggest that cortico-thalamostriatal disconnection may help to prevent seizure recurrence in individuals in whom the epileptogenic zone has been completely excised.^13^ This approach may not only prevent alteration of the healthy brain^6^ but also disrupt a reverberating network that may promote seizure recurrence.^13^

Disconnection, however, can also cause dysfunction. Recent evidence from resections has highlighted that damage to the white matter, rather than the cortex, is more likely to cause permanent neurological deficits.^57–59^ Therefore, if disconnection may be advocated on the basis of a better seizure outcome, the latter has to be balanced with the need to preserve function.^60^ Disconnection of anterior thalamic or corticostriatal projections was not associated with language, executive and memory deficits in this cohort. However, a more comprehensive neuropsychological and neuropsychiatric battery may be required to fully assess the effect of this pattern of disconnection, particularly when considering that some of these pathways, such as the anterior thalamic radiation, are critical components of the Papez circuit that mediate memory and emotion^61,62^ and their disconnection has been linked to cognitive control deficits.^36^ Accordingly, while our results suggest that disconnection should be considered together with lesion resection in frontal lobe epilepsy surgery, planned disconnection of these pathways must be tailored to each patient's history, neuropsychological data and expectations, prioritizing the need to offer the optimal balance between seizure control and preservation of function.

### Limitations

This study has a number of limitations. Our cohort of 47 patients is heterogeneous in terms of aetiology, and confirmation of our results is needed in a larger cohort, although this is the largest sample with advanced imaging analysis that has assessed long-term seizure outcome in frontal lobe epilepsy. Similarly, different pathologies may impact differently on brain reorganization,^45^ and future work has to disentangle cognitive alterations specific to distinct etiological and pathological entities.

## Conclusions

Epilepsy is increasingly considered to be a network disorder. In this study, we show that disconnection beyond ablation of the epileptogenic zone may prevent delayed seizure recurrence, and cortico-thalamic and cortico-striatal disconnection may play a critical role in retaining seizure freedom in those patients with complete resection of the epileptogenic lesion. These connections may constitute an epileptogenic network common to patients with frontal lobe epilepsy, irrespective of lesion location. Tailored disconnection of this network, taking into account the individual functional status and cognitive reserve, may complement extant surgical resection practices and lead to improved seizure outcomes.

## Supplementary Material

awad085_Supplementary_Data
