# Supplementary material for: Thalamostriatal disconnection underpins long-term seizure freedom in frontal lobe epilepsy surgery
Source: Brain. 2023 Apr 17;146(6):2377–88. doi: 10.1093/brain/awad085 (PMC10232243; doi:10.1093/brain/awad085)
Supplement: awad085_Supplementary_Data [file awad085_supplementary_data.pdf]

Supplementary Material

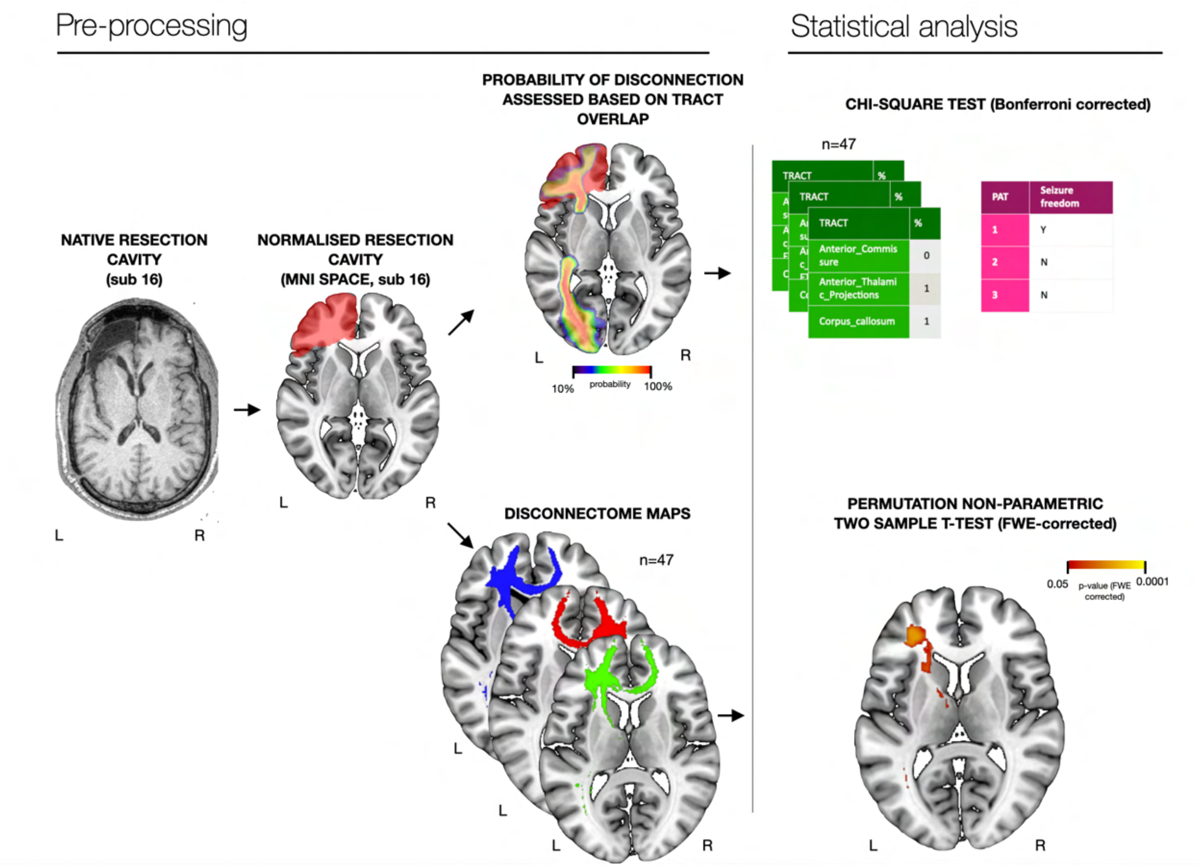

Supplementary Fig. 1 Pre-processing and analysis of voxel- and atlas-based disconnectome

A flow-chart of voxel- and atlas-based analysis is shown. First the postoperative T1 and related resection cavity is normalised to the MNI space. Then the normalised resection cavity is used to calculate probability of disconnection using Tractotron, and disconnectome maps. Then a chi-square test and a voxel-based, permutation non-parametric two sample t-test are performed.

Atlas-based analysis

Patient	Anterior thalamic radiation	Anterior fronto-striatal projection
1	Preserved	Preserved
2	Disconnected	Disconnected
3	Disconnected	Disconnected
4	Preserved	Preserved

5	Preserved	Preserved
6	Preserved	Preserved
7	Disconnected	Disconnected
8	Disconnected	Disconnected
9	Preserved	Preserved
10	Disconnected	Preserved
11	Preserved	Preserved
12	Preserved	Preserved
13	Preserved	Preserved
14	Disconnected	Disconnected
15	Preserved	Preserved
16	Disconnected	Disconnected
17	Preserved	Preserved
18	Preserved	Preserved
19	Disconnected	Disconnected
20	Preserved	Preserved
21	Disconnected	Disconnected
22	Disconnected	Preserved
23	Preserved	Preserved
24	Preserved	Preserved
25	Preserved	Preserved
26	Disconnected	Disconnected
27	Preserved	Preserved
28	Disconnected	Disconnected
29	Preserved	Preserved
30	Preserved	Preserved
31	Preserved	Preserved
32	Preserved	Preserved
33	Disconnected	Disconnected
34	Preserved	Preserved
35	Preserved	Preserved
36	Disconnected	Disconnected
37	Preserved	Disconnected
38	Preserved	Preserved
39	Preserved	Preserved
40	Preserved	Preserved
41	Preserved	Preserved
42	Preserved	Preserved
43	Preserved	Preserved
44	Disconnected	Disconnected
45	Disconnected	Disconnected
46	Preserved	Preserved
47	Preserved	Preserved

Supplementary Table 1 Atlas-based disconnection values of significant tracts using Tractotron in the different patients

There was no association between thalamostriatal disconnection and seizure outcome in the different time-points in partial resections (3 years: anterior thalamic radiation ($\chi^2(1) = 1.945$, $p_{corrected} = 0.163$); corticostriatal disconnection ($\chi^2(1) = 0.678$, $p_{corrected} = 0.410$); 5 years: anterior thalamic radiation ($\chi^2(1) = 0.417$, $p_{corrected} = 0.519$); corticostriatal disconnection ($\chi^2(1) = 0.085$, $p_{corrected} = 0.770$))

Tractography-based analysis

Patient	Hemisphere	ATR_pre	ATR_post	% ATR disconnected	FST_pre	FST _post	% FST disconnected
2	Left	2369	2096	12.0%	1197	1197	0.0%
3	Left	5920	5918	0.0%	1544	1544	0.0%
9	Left	1756	1326	24.5%	512	490	4.3%
10	Left	1314	677	48.5%	984	909	7.6%
11	Left	2266	1102	51.4%	1108	316	71.5%
14	Left	751	417	44.5%	1048	1025	2.2%
19	Left	820	75	90.9%	8870	3011	66.1%
20	Left	1138	1062	6.7%	695	672	3.3%
23	Left	1261	1247	1.1%	922	922	0.0%
25	Left	1408	872	38.1%	836	523	37.4%
33	Right	786	637	19.0%	1427	1368	4.1%
34	Right	1190	923	22.4%	1133	1099	3.0%
35	Right	740	686	7.3%	386	337	12.7%
36	Right	1717	433	74.8%	1629	501	69.2%
38	Right	1052	1047	0.5%	529	470	11.2%
41	Right	1043	288	72.4%	1341	11	99.2%
46	Left	1194	864	27.6%	199	151	24.1%

Supplementary Table 2 Native tractography-based disconnectome analysis and percentages of disconnected tract streamlines

Relevant tracts from previous disconnectome analyses are dissected in a subgroup of 17 patients who also underwent preoperative tractography. Number of streamlines before (_pre)

and after (_post) resection is displayed. Residual tracts are shown as percentage. ATR: anterior thalamic radiation; FST; fronto-striatal projections

Patient	Anterior thalamic radiation	Anterior fronto-striatal projection
	**Streamlines**	
	2995 ± 778	460 ± 285
	**% of disconnection**	
1	0.51	0.37
2	1.00	0.93
3	1.00	0.98
4	0.46	0.46
5	0.73	0.61
6	0.56	0.88
7	1.00	0.99
8	1.00	0.98
9	0.84	0.83
10	1.00	0.94
11	0.78	0.67
12	0.49	0.23
13	0.97	0.85
14	1.00	0.98
15	0.83	0.80
16	1.00	0.99
17	0.31	0.18
18	0.52	0.29
19	1.00	1
20	0.83	0.66
21	0.49	0.14
22	0.67	0.78
23	0.67	0.82
24	0.62	0.38
25	0.97	0.28
26	0.46	0.66
27	0.15	0.78
28	0.91	0.95
29	1.00	0.90
30	1.00	1
31	0.79	0.51
32	0.82	0.72
33	0.99	0.22

34	1.00	0.71
35	0.66	0.22
36	1.00	1
37	0.81	0.44
38	0.93	0.65
39	0.51	0.62
40	0.98	0.91
41	0.36	0.46
42	0.10	0.03
43	1.00	0.99
44	0.58	0.34
45	0.90	0.56
46	0.64	0.20
47	0.68	0.67

Supplementary Table 3 Normative tractography-based disconnectome analysis and percentages of disconnected tract streamlines

Normative dataset

Number of streamlines disconnected in complete versus partial resections

There was no significant difference in the number of streamlines disconnected in complete vs partial resections: anterior thalamic radiation (t(25.512)= 1.199; p=0.246); fronto-striatal projections (t(45)= 0.582; p= 0.563)

There was no association between thalamostriatal disconnection and seizure outcome in the different time-points in partial resections (3 years: anterior thalamic radiation (t(16)= 1.117, $p_{corrected} = 0.280$); corticostriatal disconnection (t(16)= 1.199, $p_{corrected} = 0.248$); 5 years: anterior thalamic radiation ($\chi^2(1) = 0.417$, $p_{corrected} = 0.519$); corticostriatal disconnection ($\chi^2(1) = 0.085$, $p_{corrected} = 0.770$))

Neuropsychological evaluation and disconnection analysis

We investigated whether disconnection of anterior thalamic/fronto-striatal projections was associated with deficits in three domains (language, executive, memory). For each analysis

there was no significant neuropsychological deficit in these domains associated with anterior thalamic or anterior fronto-striatal disconnection at 1 year.

Atlas-based analysis

Anterior thalamic radiation

Graded naming task: no patient of our cohort was significantly worse at follow-up

Phonemic fluency: ($\chi^2(1) = 0.120$, $p_{corrected} = 0.729$)

Semantic fluency: ($\chi^2(1) = 0.285$, $p_{corrected} = 0.593$)

BMIPM: ($\chi^2(1) = 0.003$, $p_{corrected} = 0.957$)

Anterior cortico-striatal projection

Graded naming task: no patient of our cohort was significantly worse at follow-up

Phonemic fluency: ($\chi^2(1) = 0.295$, $p_{corrected} = 0.587$)

Semantic fluency: ($\chi^2(1) = 0.036$, $p_{corrected} = 0.850$)

BMIPM: ($\chi^2(1) = 0.012$, $p_{corrected} = 0.912$)

Native tractography-based analysis

Anterior thalamic radiation

Graded naming task: no patient of our cohort was significantly worse at follow-up

Phonemic fluency: ($t(12)= -0.948$; $p=0.362$)

Semantic fluency: ($t(13)= -2.126$; $p=0.053$)

BMIPM: ($t(12)=-1.404$; $p=0.186$)

Anterior cortico-striatal projection

Graded naming task: no patient of our cohort was significantly worse at follow-up

Phonemic fluency: ($t(12)= -0.384$; $p=0.708$)

Semantic fluency: ($t(13)= 0.386$; $p=0.386$)

BMIPM: ($t(2.130)=-2.079$; $p=0.165$)

Normative tractography-based analysis

Anterior thalamic radiation

Graded naming task: no patient of our cohort was significantly worse at follow-up

Phonemic fluency: ($t(35)= -0.810$; $p=0.423$)

Semantic fluency: ($t(36)= 1.140$; $p=0.262$)

BMIPM: (t(33)=0.444; p=0.660)

Anterior cortico-striatal projection
Graded naming task: no patient of our cohort was significantly worse at follow-up
Phonemic fluency: (t(35)= -0.150; p=0.865)
Semantic fluency: (t(36)= 1.085; p=0.285)
BMIPM: (t(33)=0.730; p=0.471)

Descriptive cases

Descriptive case 1
28 years old, right-handed gentleman (Pat. 36 of our series), developed convulsions at night before the age of five.

Complex motor seizures involved stiffening and breath hold, then he would become red in the face, his fists would clench and he would sit up in bed. He then would exhale and try to get out of bed. He will say that he needed to go to the toilet but was usually not aware of this. The seizures occurred 2-3 nights per week, with clustering of 3-4 seizures per night. Before surgery he was taking Levetiracetam 1000 mg twice a day and Zonisamide 150 mg twice a day.

There was a strong family history for epilepsy affecting his father, grandfather and his grandfather's brothers. He was independent - although a parent slept in his room at night due to his seizures. He delayed his university education due to his epilepsy. He was unemployed. Neurological examination was unremarkable.

Scalp video-EEG telemetry recorded hyperkinetic seizures, with rocking movements of the trunk, tonic flexion of both arms, tonic extension of the left leg, jaw clenching, and kicking movements of both legs. The ictal EEG showed diffuse attenuation and EMG/movement artefacts at the onset. After ~20 seconds, a right frontal pattern emerged in all seizures. The interictal EEG showed frequent epileptiform features over the right frontopolar area. There were widespread non-specific markers (slowing) of dysfunction in both frontotemporal areas. MRI did not show abnormalities (Suppl. Fig S2).

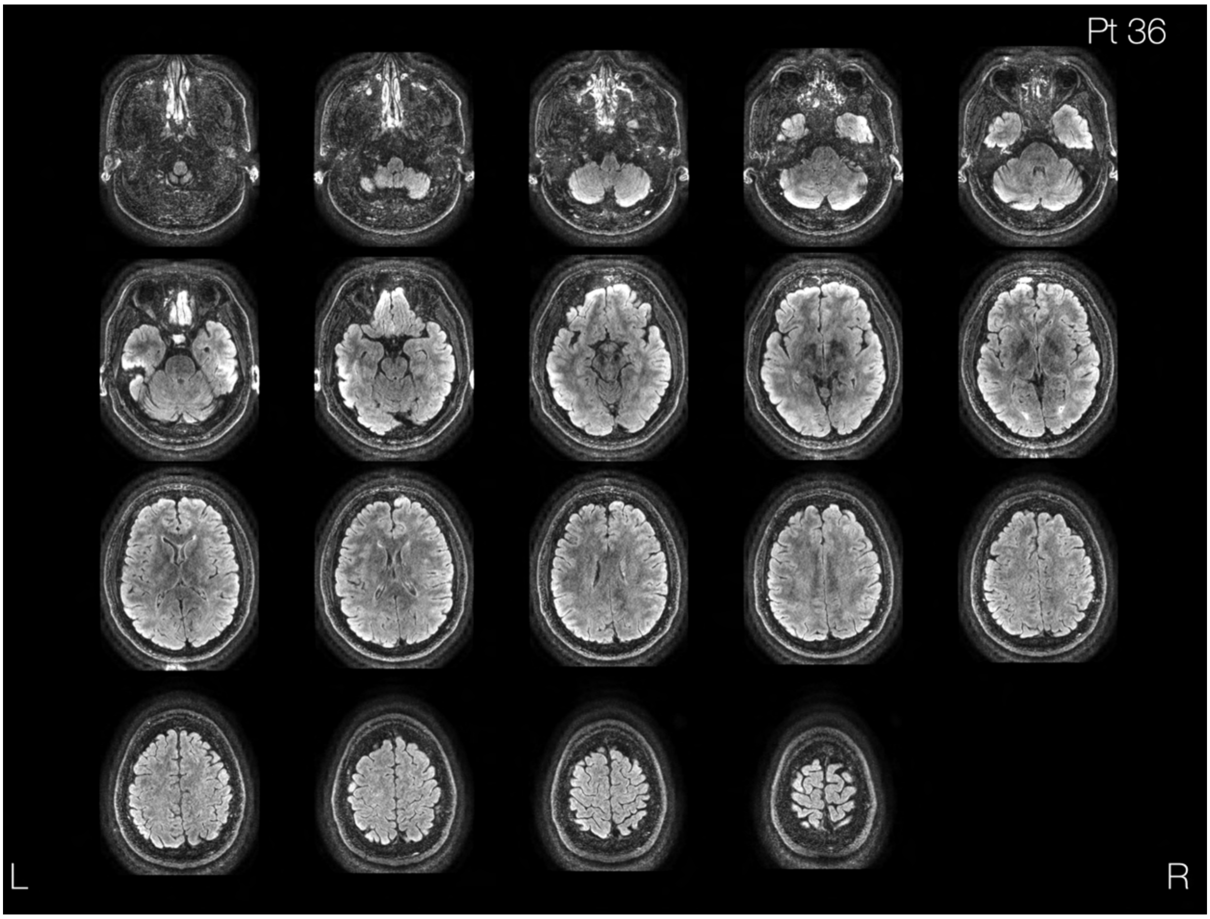

Suppl. Fig 2 Preoperative volumetric FLAIR MRI

Language fMRI showed left hemispheric language dominance. FDG-PET showed right medial frontal lobe hypometabolism (Suppl. Fig S3).

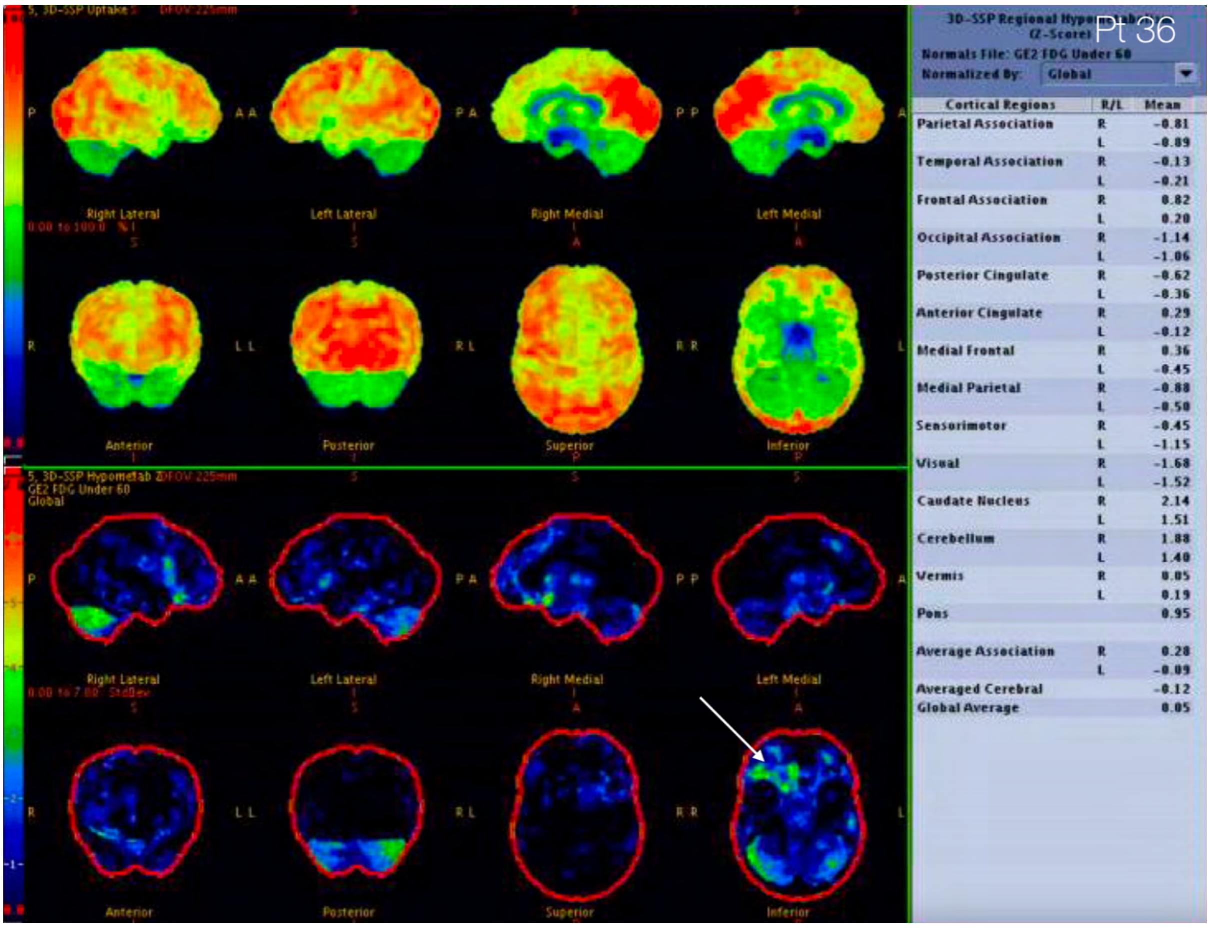

Suppl. Fig 3 Preoperative FDG-PET.

Right orbitofrontal hypometabolism is shown, highlighted by a white arrow

Neuropsychology showed mild under-functioning from his estimated mid-average potential. Reasoning skills were weak, working and episodic memory were inefficient. Expressive language and perceptual functions were intact. Difficulties were elicited on tests tapping attention and executive functions. His cognitive profile and test behaviour provided support for frontal lobe involvement but with no strong lateralization. Neuropsychiatry evaluation did not note any disorder. The presumed cortical epileptogenic zone was thought to be in the right orbitofrontal cortex and it was recommended to perform invasive EEG recordings to identify and delineate the epileptogenic zone.

The patient underwent stereotactic implantation of 8 electrodes targeting the amygdala, hippocampus, middle frontal gyrus to anterior cingulum, pre-supplementary motor area (preSMA), supplementary motor area (SMA), inferior frontal gyrus (IFG), middle frontal gyrus (MFG) and lateral (LOF) and medial orbitofrontal areas (MOF) (Suppl. Figures S4a-b-c).

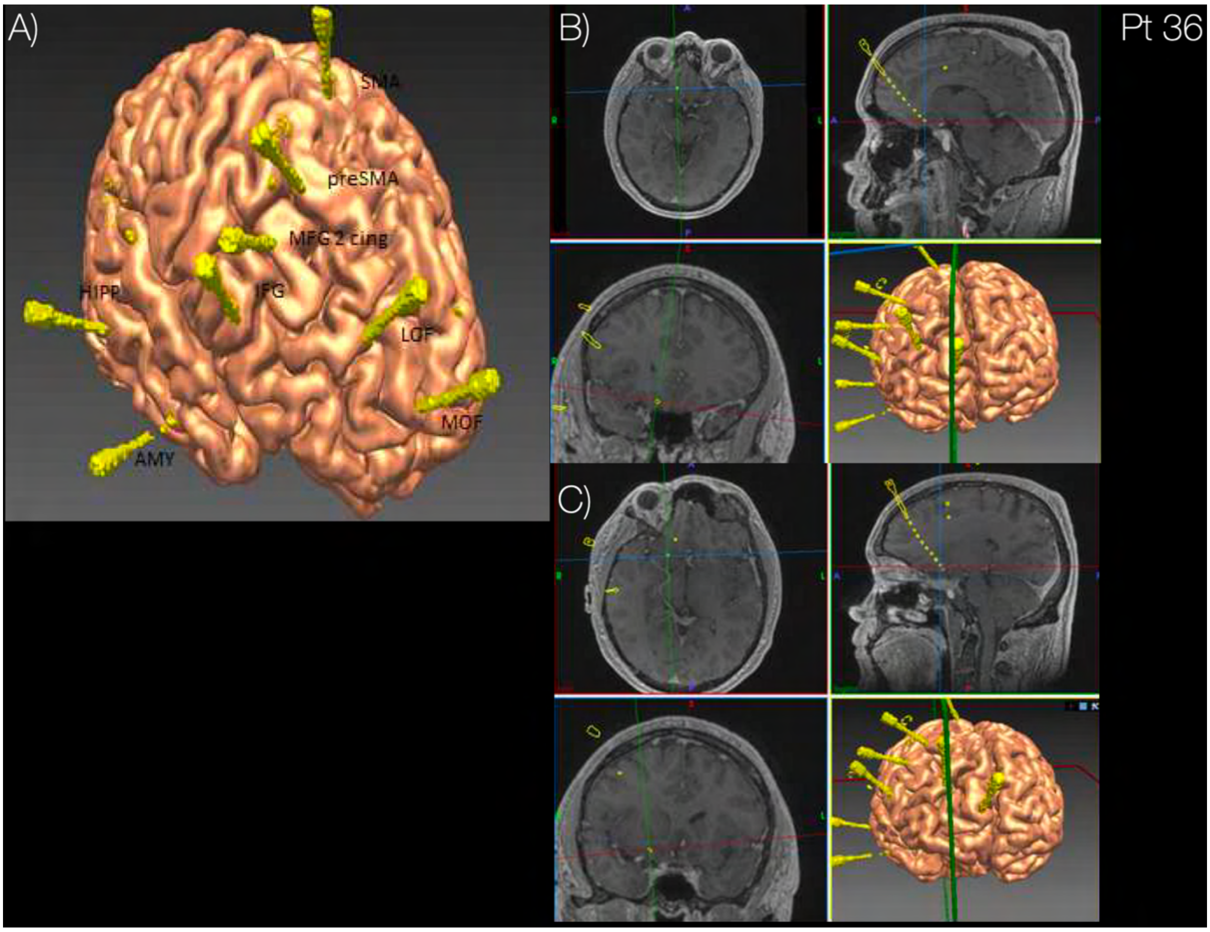

Suppl. Fig 4 Invasive recording using SEEG

a)3D visualisation using EpiNav b) Mesial orbitofrontal electrode (centred on the cross) c) Lateral orbitofrontal electrode (centred on the cross)

Legend: Pre-supplementary motor area (preSMA), supplementary motor area (SMA), inferior frontal gyrus (IFG), and lateral (LOF) and medial orbitofrontal areas (MOF), amygdala (AMY) hippocampus (Hipp), middle frontal gyrus, (MFG)

The presurgical planning was performed using an in-house software, EpiNav. Interictal spikes were recorded from hippocampal and orbitofrontal contacts. The patient had four stereotypical seizures during sleep: common seizure symptoms were prominent proximal leg movements (flexion/extension and ab/adduction), abdominal "crunching", and manual automatisms. This was followed by axial and limb tonic stiffening resulting in transient apnoea. Loss of awareness without postictal language problems was demonstrated in all seizures.

Electrographically, the first sustained ictal rhythm was seen over the orbitofrontal contacts LOF 1, then spreading over further contacts of electrodes MOF and LOF (Suppl. Fig S5), lateral contacts of MFG, IFG, and the SMA contacts.

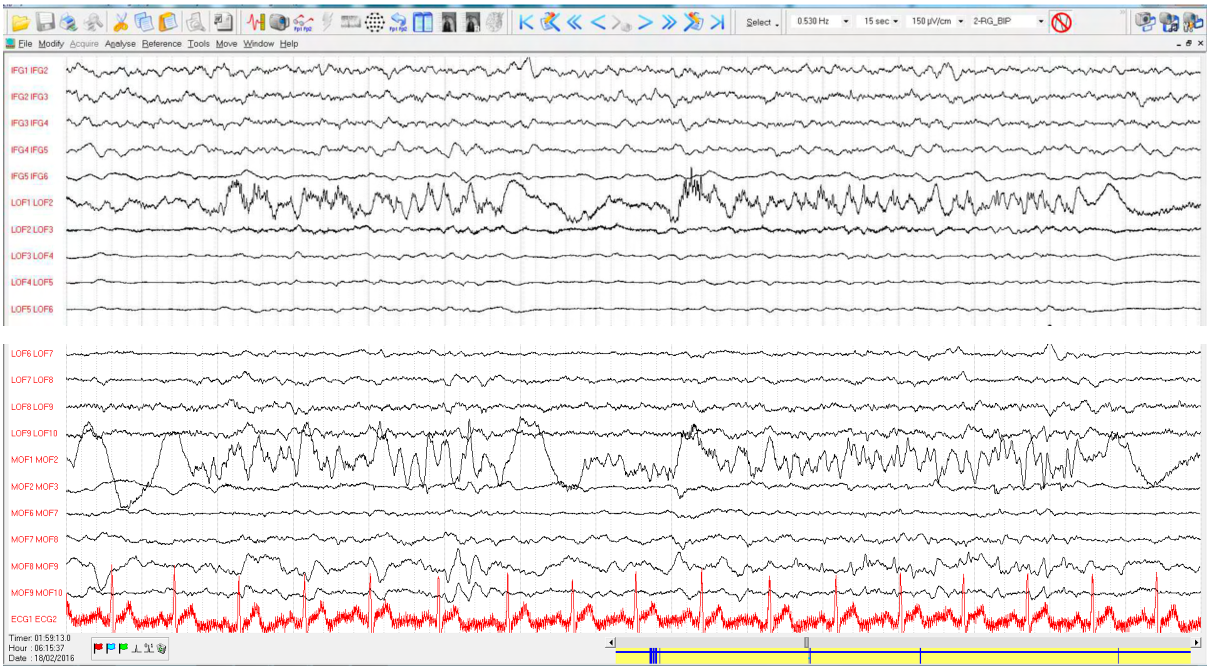

Suppl. Fig 5 Invasive recording using SEEG

Intermittent bursts of fast activity followed by theta activity leading up to a seizure involving lateral (LOF) and medial (MOF) orbitofrontal cortex

Considering these results, the onset zone was thought to be in the right orbitofrontal cortex.

The patient underwent resection of the right orbitofrontal cortex, gyrus rectus, the frontal pole and the pars orbitalis (Suppl. Fig S6). Atlas-based disconnection analysis showed that anterior thalamic and fronto-striatal projections were disconnected.

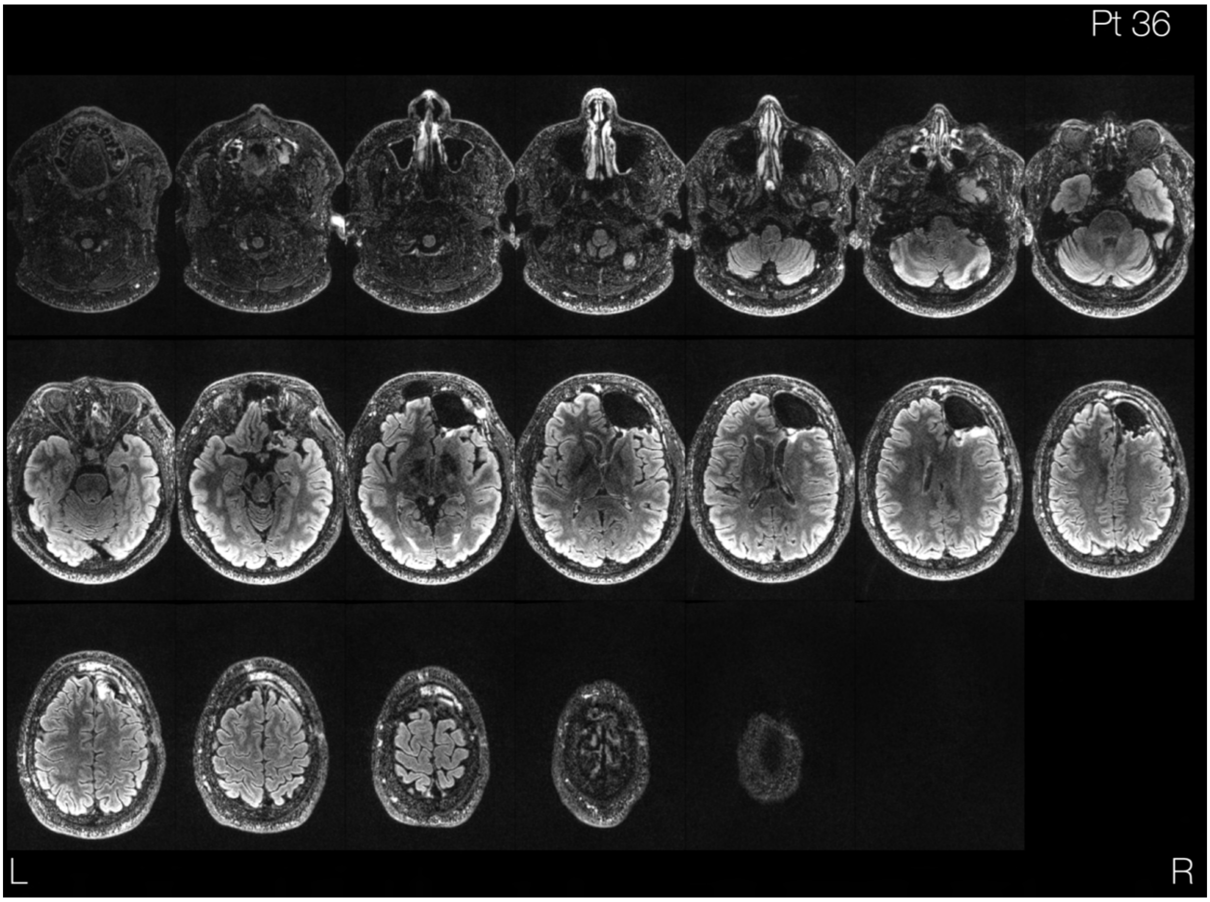

Suppl. Fig 6 Postoperative FLAIR MRI

Analysis of the preoperative tractography before and after surgery showed disconnection of the anterior thalamic radiation (75%) and fronto-striatal projections (69%) (Suppl Fig. 7).

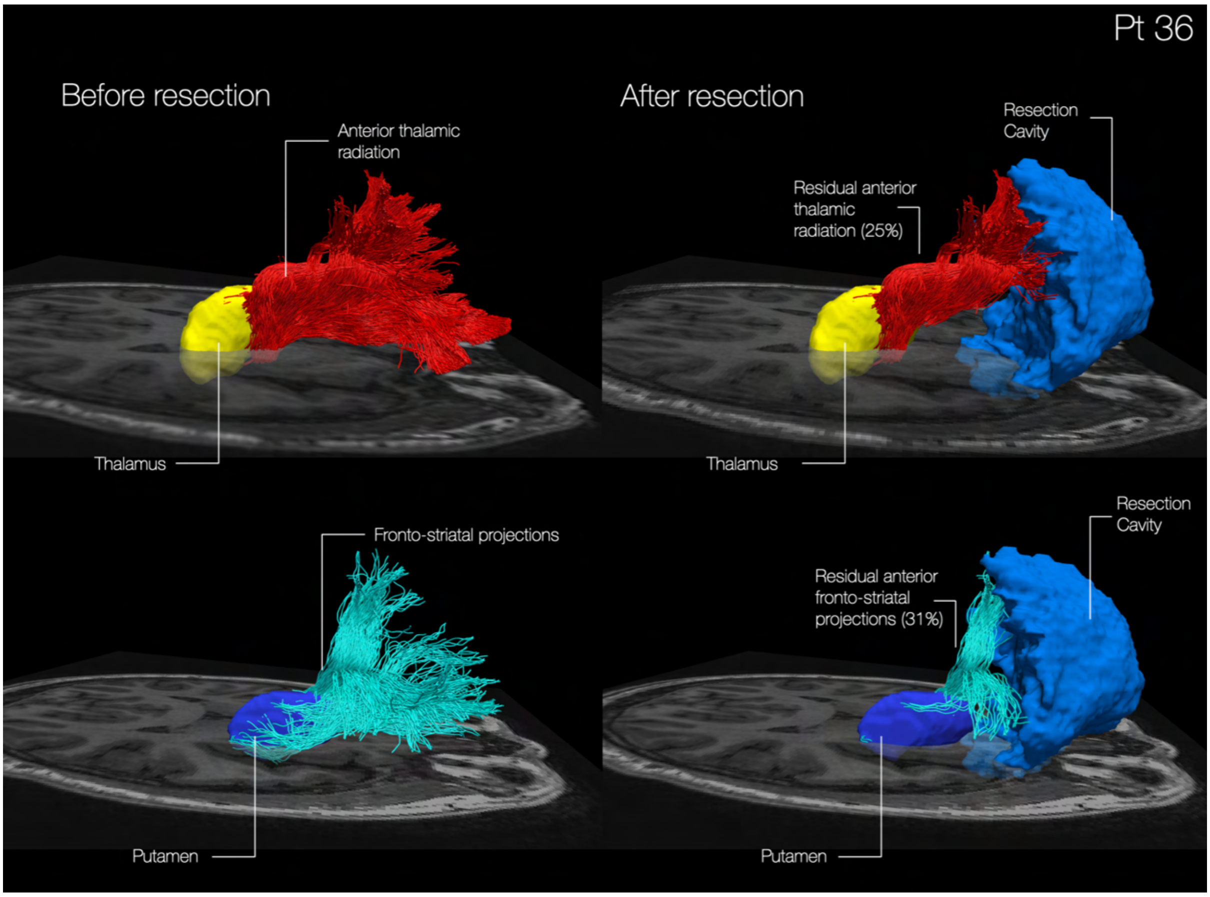

Suppl. Fig 7 Anterior thalamic and fronto-striatal disconnection

75% of the anterior thalamic radiation and 69% of the anterior fronto-striatal projections were disconnected during surgery

After surgery, the patient has remained seizure free, until present, four years after surgery. Histology showed cortical dysplasia, with occasional isolated enlarged neurones with excess of lipofuscin but without clear characterisation. He currently takes Phenytoin 500mg nocte Lamotrigine 350mg twice a day, Clobazam 10mg mane / 5 mg nocte.

Demonstration case 2

A 53-year old, right-handed lady (Pat. 15 of our series). Seizure onset was at 30 years. Initially she had focal unaware seizures, from the age of 31 she developed focal to bilateral tonic-clonic seizures. Seizures were refractory to medical treatment. She had automotor seizures with loss of awareness: she would stare blankly into space and may talk and laugh inappropriately. These were combined with occasional orofacial automatisms. Seizures lasted 45-50 seconds, with

post-ictal dysnomia/word-finding difficulties. The frequency was 1-3 per week. Focal to bilateral tonic-clonic seizures occurred 2-3 times per year. She had no family history for epilepsy. She worked as a book-keeper until her forties. Presurgically, she took Lamotrigine 300 mg twice daily. Neurological examination was unremarkable.

Several seizures were recorded during video-EEG telemetry. The majority demonstrated behavioural arrest, and on 1-2 occasions she had intelligible but out-of context speech (jargon aphasia). The EEG showed interictal bilateral fronto-temporal sharp waves. Amplitude was higher on the left side. Ictal onset was bifrontotemporal and non-lateralising. Brain MRI showed a non-enhancing lesion isointense in T1-weighted sequences and hyperintense in T2-weighted sequences in the left subfrontal regions adjacent with the gyrus rectus. The appearance was suggestive of a dysembryoplastic neuroepithelial tumour, with the possibility of a meningioma (Suppl. Fig S8).

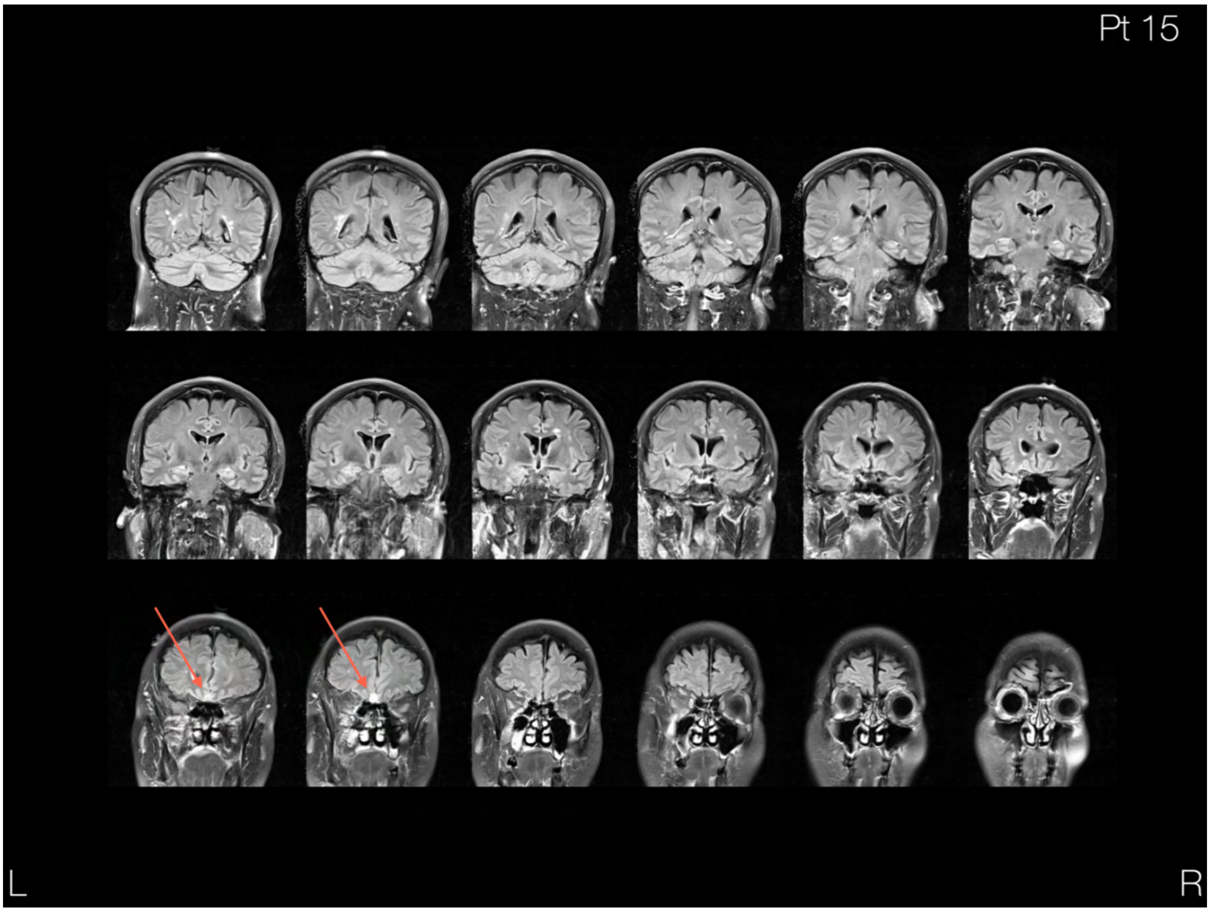

Suppl. Fig 8 Preoperative FLAIR-weighted imaging

A left mass at the level of the gyrus rectus is shown, highlighted with a red arrow

Language fMRI showed she was left-hemisphere dominance. MEG showed spike clusters in the left basal temporal, left orbitofrontal and more widespread left frontotemporal region (Suppl. Fig S9).

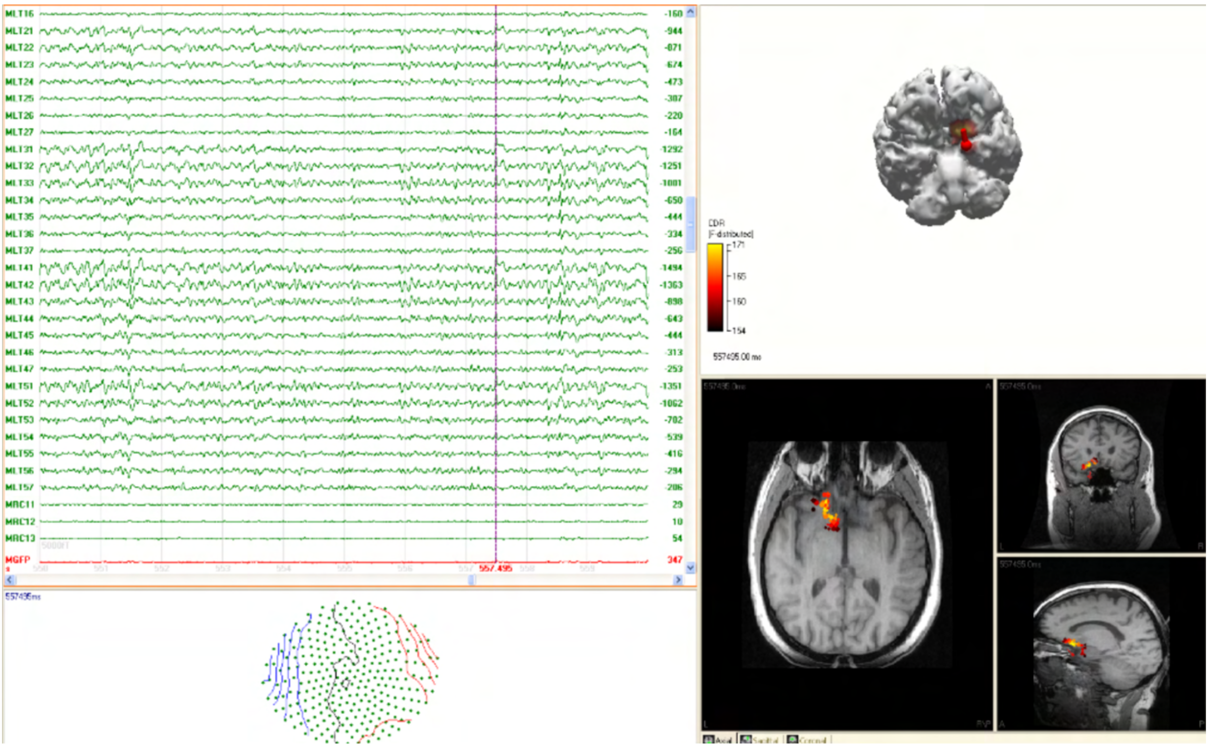

Suppl. Fig 9 Magnetoencephalography results

MEG results with spike clusters and the associated functional region in the left orbitofrontal cortex is shown

Neuropsychological assessment indicated that her current verbal intellectual capacity was above average and at her optimal level. Overall, her cognitive profile was good with slight underfunctioning, without lateralising or localising features. Neuropsychiatric assessment noted a tendency to low mood and she was counselled for the risks of surgery. The MDT considered most likely seizures arose from the left orbitofrontal cortex. The precise nature of the left orbitofrontal lesion was considered unclear.

Invasive recordings were planned through seven SEEG electrodes covering the left frontal pole, left gyrus rectus, left middle orbitofrontal gyrus, left lateral orbitofrontal gyrus, left temporal pole, left amygdala and left hippocampus (Suppl. Figure S10).

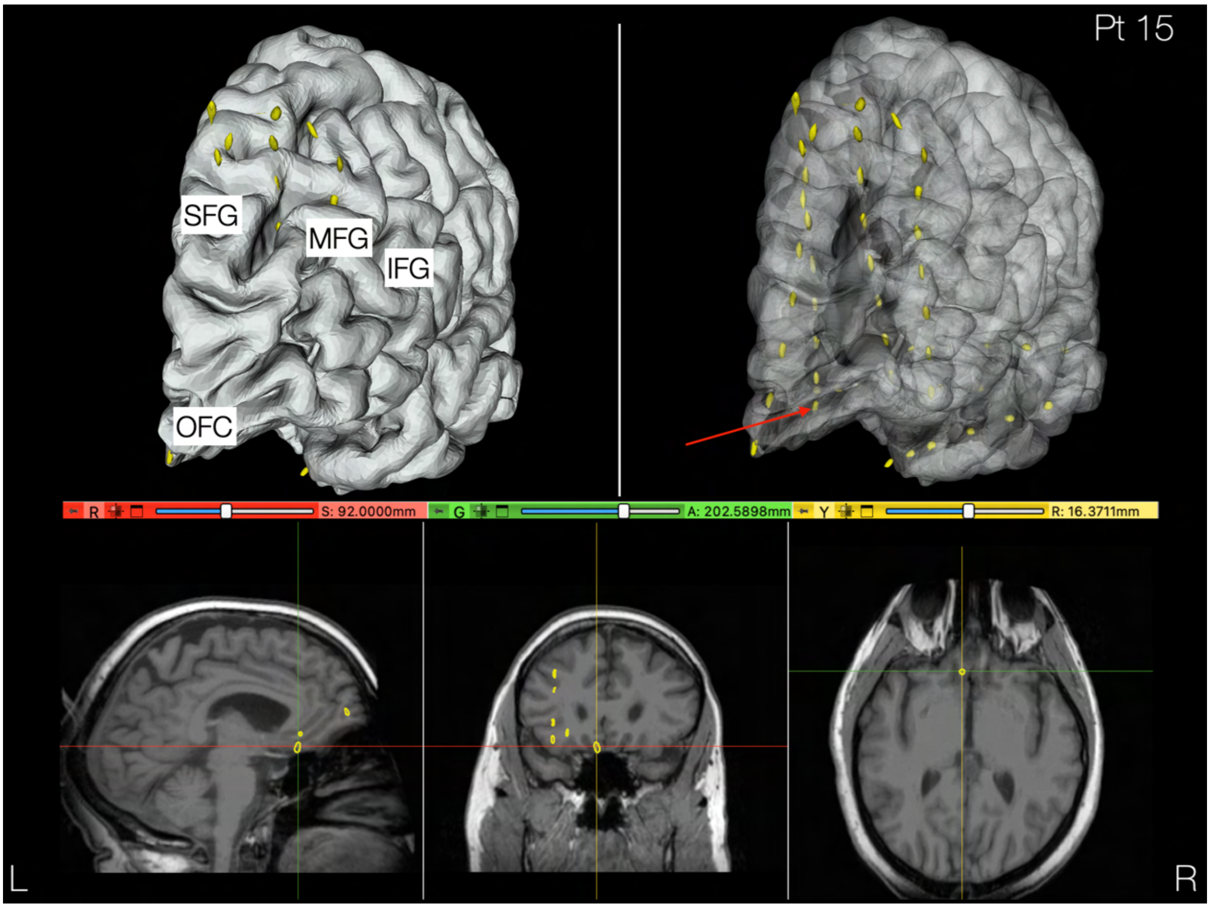

Suppl. Fig 10 Invasive recording using SEEG – 3D planning

Upper: 3D distribution of the depth electrodes to cover left frontal pole, the left gyrus rectus, the left middle orbitofrontal gyrus, the left lateral orbitofrontal gyrus, the left temporal pole, the left amygdala and the left hippocampus (left: cortical surface with superficial contacts; right: shaded cortical surface with deep contacts location). Contact in proximity of the lesion is shown with a red arrow. Sectional anatomy and contact location are shown in the lower panel.

Multiple seizures were recorded. These started with crying or sobbing and then would develop into automotor seizures with right hand tremor and lip smacking. Post-ictally she showed comprehension disorders both for naming objects and for commands. All seizures had a stereotypical EEG pattern involving the orbitofrontal cortex. Seizure onset was considered to be on the left orbitofrontal cortex (Suppl. Figure S10-S11).

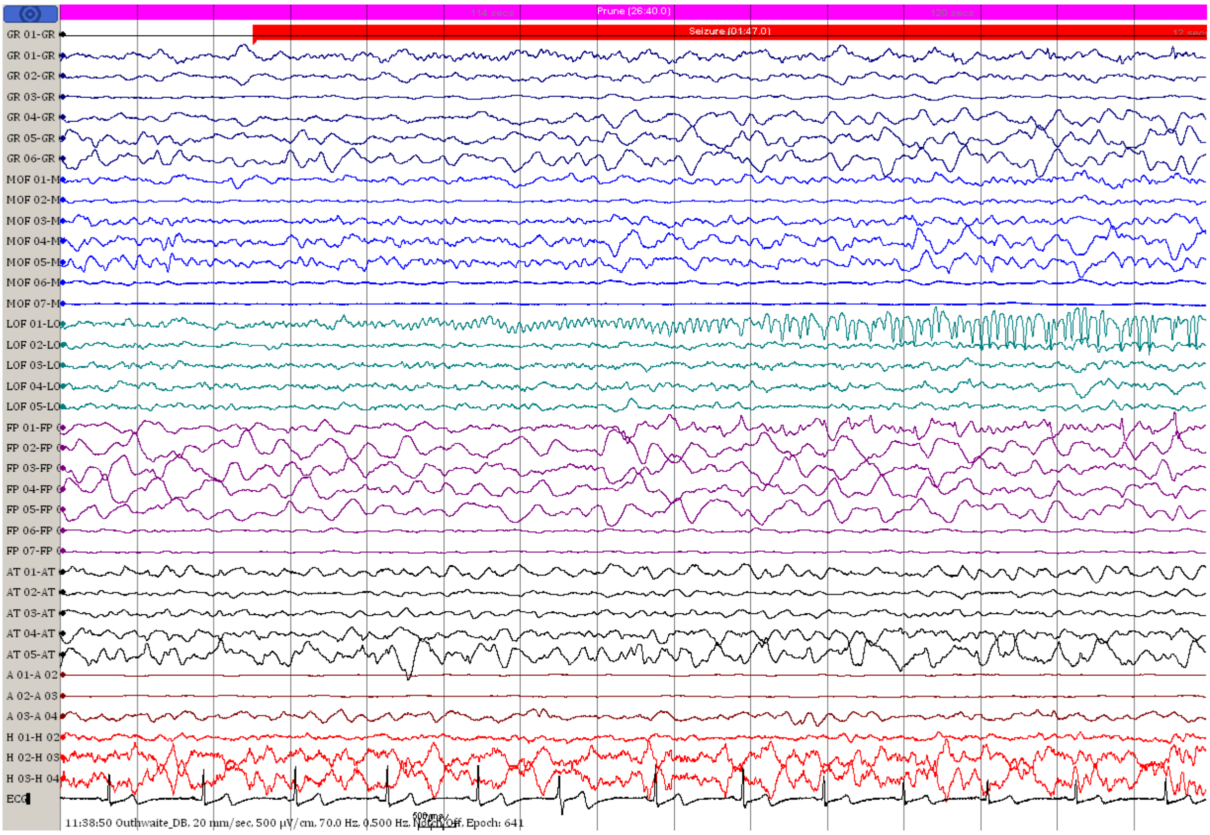

Suppl. Fig 11 Invasive recording using SEEG (bipolar montage)

Rhythmic activity evolving in amplitude and frequency was seen over contact LOF 01.

The patient underwent resection under awake surgery. No intraoperative seizures were recorded. Postoperative MRI showed a complete resection of the lesion and excision of the left anterior gyrus rectus and orbitofrontal cortex (Suppl. Fig 12).

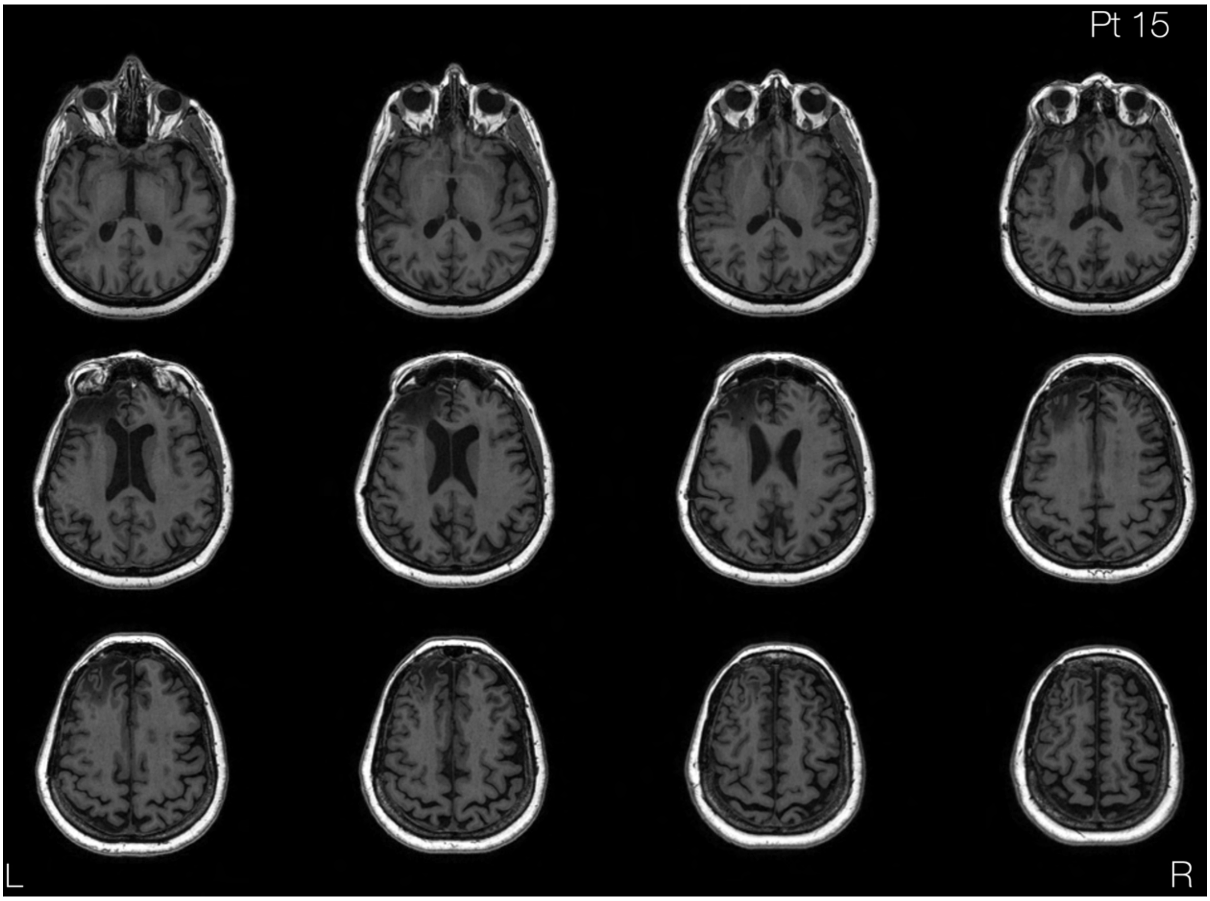

Suppl. Fig 12 Postoperative T1-weighted MRI

After surgery, the patient developed mild anomia and was followed up by speech and language therapists for rehabilitation. She was not seizure free, however, frequency decreased postoperatively to one seizure per week. Histology showed a dysembryoplastic neuroepithelial tumour (WHO I). On the longer term, the seizures worsened to twice a week despite medication changes. At the neuropsychological review 5 years after surgery word-finding difficulties had resolved, however, but there were declines on measures of working memory and verbal fluency. 10 years after surgery, she had complex motor seizures twice per week, with one focal to bilateral tonic-clonic seizure per week, mostly arising during sleep.
